# Interaction between Microbes and Host in Sow Vaginas in Early Pregnancy

**DOI:** 10.1128/msystems.01192-22

**Published:** 2023-02-07

**Authors:** Xupeng Zang, Wenjing Wang, Shengchen Gu, Ting Gu, Huaqiang Yang, Enqin Zheng, Zheng Xu, Sixiu Huang, Zicong Li, Gengyuan Cai, Linjun Hong, Zhenfang Wu

**Affiliations:** a National Engineering Research Center for Breeding Swine Industry, College of Animal Science, South China Agricultural University, Guangzhou, China; b Guangdong Provincial Key Laboratory of Agro-Animal Genomics and Molecular Breeding, College of Animal Science, South China Agricultural University, Guangzhou, China; c National Local Joint Engineering Research Center of Livestock and Poultry, South China Agricultural University, Guangzhou, China; d State Key Laboratory for Conservation and Utilization of Subtropical Agro-bioresources, Guangzhou, China; The University of Maine

**Keywords:** developing microbiota, early pregnancy, pig, vaginal microbiota

## Abstract

Extensive research has explored the causes of embryo losses during early pregnancy by analyzing interaction mechanisms in sows’ uterus, ignoring the importance of the lower reproductive tract in pregnancy development regulation. Despite recent progress in understanding the diversity of vaginal microbes under different physiological states, the dynamic of sows’ vaginal microbiotas during pregnancy and the interaction between vaginal microbes and the host are poorly understood. Here, we performed a comprehensive analysis of sows’ vaginal microbial communities in early pregnancy coupled with overall patterns of vaginal mucosal epithelium gene expression. The vaginal microbiota was analyzed by 16s rRNA or metagenome sequencing, and the vaginal mucosal epithelium transcriptome was analyzed by RNA sequencing, followed by integration of the data layers. We found that the sows’ vaginal microbiotas in early pregnancy develop dynamically, and there is a homeostasis balance of *Firmicutes* and *Proteobacteria*. Subsequently, we identified two pregnancy-specific communities, which play diverse roles. The microbes in the vagina stimulate the epithelial cells, while vaginal epithelium changes its structure and functions in response to stimulation. These changes produce specific inflammation responses to promote pregnancy development. Our findings demonstrate the interaction between microbes and host in the sow vagina in early pregnancy to promote pregnancy development, meanwhile providing a reference data set for the study of targeted therapies of microbial homeostasis dysregulation in the female reproductive tract.

**IMPORTANCE** This work sheds light on the dynamics of the sow vaginal microbiotas in early pregnancy and its roles in pregnancy development. Furthermore, this study provides insight into the functional mechanisms of reproductive tract microbes by outlining vaginal microbe-host interactions, which might identify new research and intervention targets for improving pregnancy development by modulating lower reproductive tract microbiota.

## INTRODUCTION

Due to similar anatomical and physiological characteristics to humans, pigs not only provide high-quality proteins for animal husbandry, but also play an indispensable role in biomedical research (1, 2). Studies have emphasized that approximately 20 to 30% of pig embryos are lost spontaneously during early pregnancy (around day 12 to 30 of pregnancy), which severely limits the litter size (3, 4). Although the exact cause for embryo losses is yet to be defined, abundant studies have focused on the regulation mechanism in the sow uterus, including the interaction between embryos, uterine luminal fluid, and endometrium (5–7), while ignoring the importance of the lower reproductive tract in pregnancy development.

Previous studies have shown that there is a wide range of microbiotas in mammal vaginas, which dynamically change with the species and physiological period (8–11). Recent progress in analyzing the gene sequence of microbes in the vagina provide a comprehensive understanding of the microbial classes and the diverse ecological states of niches (12, 13). Moreover, the interaction between these commensal microbes and their colonized hosts is essential for maintaining homeostasis and causing disease (14). There is growing evidence that the microbiota from the vagina plays a key role in female reproduction and health. For women, the vaginal microbiota shifts to be dominated by lactobacilli during early pregnancy (15, 16), but after 24 weeks of pregnancy, the abundance of lactobacilli gradually decreases (17). These lactobacilli are generally believed to prevent invasion of pathogens by producing lactic acid to inhibit the growth of many other bacteria (18). Meanwhile, they could be tolerated by vaginal epithelial cells to inhibit the induction of proinflammatory cytokines (19). As one of the simplest commensal microbial communities in the human body, the dynamic nature of vaginal microbes is still complex and is crucial in host immune regulation (20). Disturbance of the vaginal microbial communities has been shown to contribute to various disease states such as bacterial vaginosis and vulvovaginal candidiasis (21–23). Unfortunately, numerous studies have reported the importance of human vaginal microbiotas, but as a vital medical-biological model for humans, the dynamic changes of the vaginal communities in sows during pregnancy are unclear, and we also do not know whether they interact with the host to regulate pregnancy development.

Hence, we present a comprehensive analysis of temporal vaginal microbiotas and microbial-associated host transcriptome in early pregnancy, revealing the diverse pattern of host-microbial interactions in the vagina at different pregnancy stages. We report the dynamic changes of sows’ vaginal microbiotas in early pregnancy, and there is a homeostasis balance of the abundance of *Firmicutes* and *Proteobacteria*. We have identified two pregnancy-specific communities, which were represented different microbial functions. The host, in turn, responds to changes in the microbiota by responding to the external stimuli and changing the expression of genes associated with matrix structure and inflammation response.

## RESULTS AND DISCUSSION

### Sows’ vaginal microbiotas in early pregnancy are enormously diverse.

**FIG 1:**
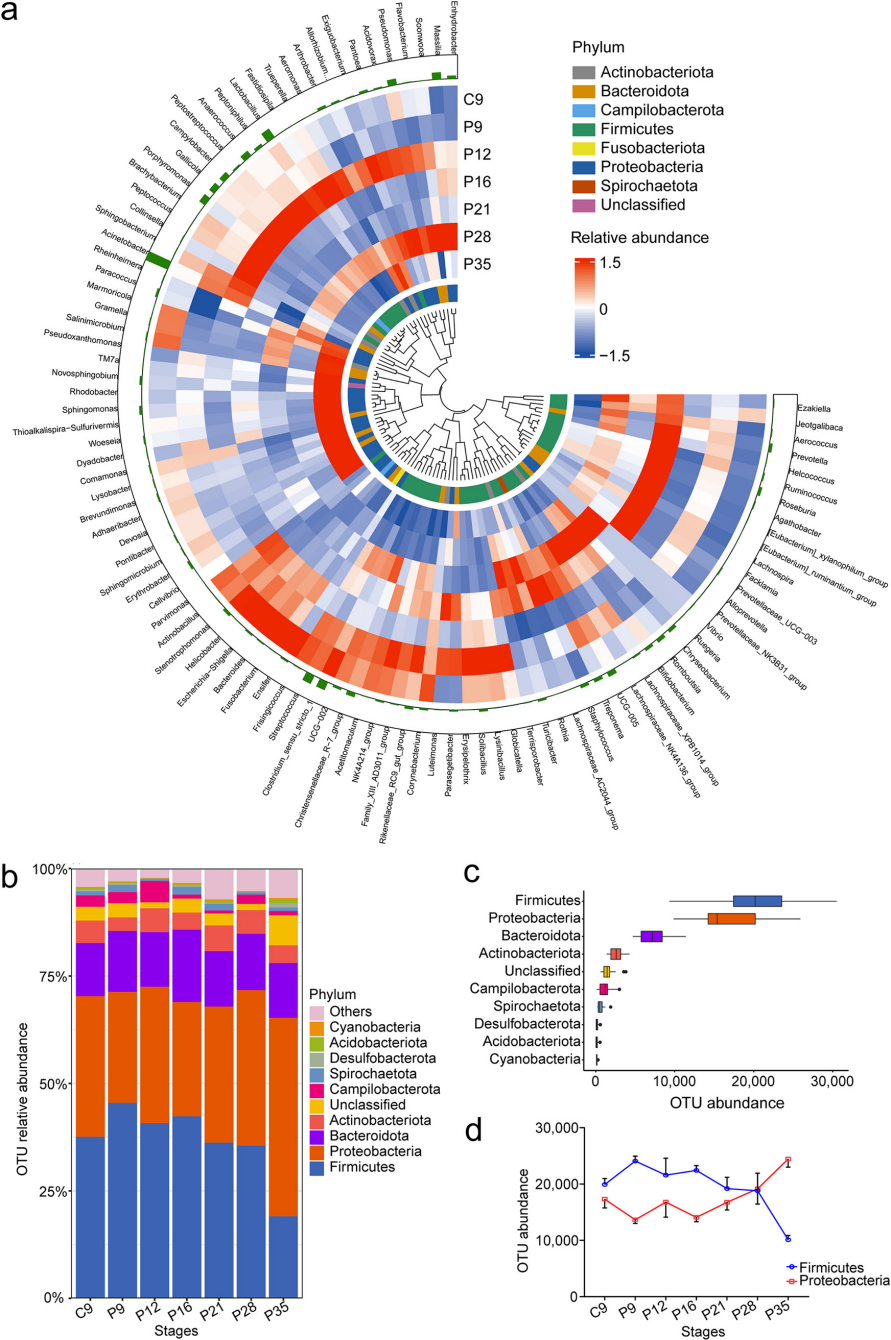
Characterization of the sow vaginal microbiome in early pregnancy. The results show significant changes in the vaginal microbiome at different stages. (a) Abundance profiles of the top 100 most abundant microbial genera at 7 different stages. The color intensity of the heatmap displays the relative abundance of each microbial genus. The length of the outer lap green bars represents the microbial genus abundance. The phylum to which the bacteria belong is indicated by different colors. (b) The most abundant bacterial phyla by the vagina. (c) Abundance of the top 10 most abundant bacterial phyla. (d) Statistical analysis (Jonckheere-Terpstra test) of the top 2 most abundant bacterial phyla showing that with the pregnancy development, the abundance of *Firmicutes* (z = −2.986, *P = *0.003) decreased significantly, while *Proteobacteria* (z = 2.786, *P = *0.005) increased significantly.

Vaginal swabs were acquired with a generalized method (see Fig. S1a and b in the supplemental material). A total of 2.75 million raw 16S rRNA gene reads were analyzed using QIIME (v.1.9.1) (24), resulting in the identification of 6,835 operational taxonomic units (OTU) at a 97% identity level (see Table S1 at https://zenodo.org/record/7537183). Sequences aligned with the SILVA 16S rRNA gene database exposed the vaginal microbiome at different early pregnancy stages in pigs. Similar to the spatiotemporal dynamics of microbiotas in most studies (8, 9, 17), the vaginal microbiotas of sows were visibly different across diverse periods (Fig. 1a and b, Fig. S2), which was confirmed by principal-component analysis (PCA) and nonmetric multidimensional scaling (NMDS) (Fig. S3a to b). Moreover, under consistent sequencing coverage (Fig. S3c; Kruskal-Wallis H test, *P* = 0.103), we did not observe any specific rules of diversity (Fig. S3d; Kruskal-Wallis H test, *P = *0.007) or richness (Fig. S3e; Kruskal-Wallis H test, *P = *0.069) with pregnancy stages, although the diversity shows significant differences between different stages. Unlike the study of infant gut microbiota by Roswall et al. (25), infant gut microbial diversity continuously increases with age. Our results are consistent with the study of Rasmussen et al. (17), the diversity of the vaginal microbial community during pregnancy does not show a specific dynamic model, which may be the result of some microbes in the vagina occupying a certain dominant.

10.1128/msystems.01192-22.1 FIG S1 (a and b) Vagina (a) sampling and (b) record flow chart. Download FIG S1, TIF file, 1.2 MB. Copyright © 2023 Zang et al. 2023 Zang et al. https://creativecommons.org/licenses/by/4.0/ This content is distributed under the terms of the Creative Commons Attribution 4.0 International license.

10.1128/msystems.01192-22.2 FIG S2 Individual composition of the vaginal microbiota in pigs at different stages of early pregnancy. At the phylum level, the most abundant bacterial group depicted for sow vagina based on the 16S rRNA gene sequence. Download FIG S2, TIF file, 1.9 MB. Copyright © 2023 Zang et al. 2023 Zang et al. https://creativecommons.org/licenses/by/4.0/ This content is distributed under the terms of the Creative Commons Attribution 4.0 International license.

10.1128/msystems.01192-22.3 FIG S3 Characterization of the sow vaginal microbiota in early pregnancy. (a) First and second principal coordinates of Euclidean distance dimensionality reduction (value in parentheses indicates the data interpretation of the dimension). Each point represents a vagina sample colored by stages. (b) NMDS analysis of vagina samples based on a Bray-Curtis dissimilarity matrix. (c to e) Average Good’s coverage and Shannon and Chao 1 diversity indexes in 7 vaginal sample sets. (f to g) Statistical analysis (Jonckheere-Terpstra test) showing that with pregnancy development, the abundance of *Bacteroidota* (z = −0.345, *P = *0.730) and *Actinobacteriota* (z = 1.435, *P = *0.151) did not change significantly. (h) Abundance dynamic of *Firmicutes* and *Proteobacteria* in different stages of pregnancy. Download FIG S3, TIF file, 2.3 MB. Copyright © 2023 Zang et al. 2023 Zang et al. https://creativecommons.org/licenses/by/4.0/ This content is distributed under the terms of the Creative Commons Attribution 4.0 International license.

When focusing on the phylum classification of these vaginal microbiotas, in accordance with recent studies of vaginal samples from gilts and pregnant sows (26), we found that *Firmicutes* and *Proteobacteria*, followed by *Bacteroidota* and *Actinobacteriota*, are the most abundant phyla in the sow vagina in early pregnancy (Fig. 1b and c). Previous studies have shown that these microbiotas are generally thought to be associated with disease suppression (27, 28). Interestingly, we found that with the development of pregnancy, the abundance of *Firmicutes* (z = −2.986, *P* = 0.003) decreased significantly, while *Proteobacteria* (z = 2.786, *P* = 0.005) increased significantly (Fig. 1d). However, for *Bacteroidota* (z = −0.345, *P* = 0.730) or *Actinobacteriota* (z = 1.435, *P* = 0.151), their abundance did not change significantly (Fig. S3f and g). Although the abundance of *Firmicutes* and *Proteobacteria* showed drastic dynamic changes, there was no significant difference in abundance for the two phyla at each stage (Kruskal-Wallis H test, *P = *0.232), and even their abundances accounted for 70% of all microbial phyla (Fig. S3h). Recent studies of the human gut microbiome also found that when the abundance of *Proteobacteria* increased significantly, there was an accompanying loss of *Firmicutes* (29, 30), implying that there would be antagonism between *Proteobacteria* and *Firmicutes*. They suppress each other’s abundance levels, thereby exerting unique biological functions for the host. Notably, the fifth most abundant phylum of sows’ vaginal microbiota has not been annotated in the existing database (Fig. 1b and c), indicating that there may be another specific microbiota playing a vital role in the vagina.

### Pregnancy-specific community types.

**FIG 2:**
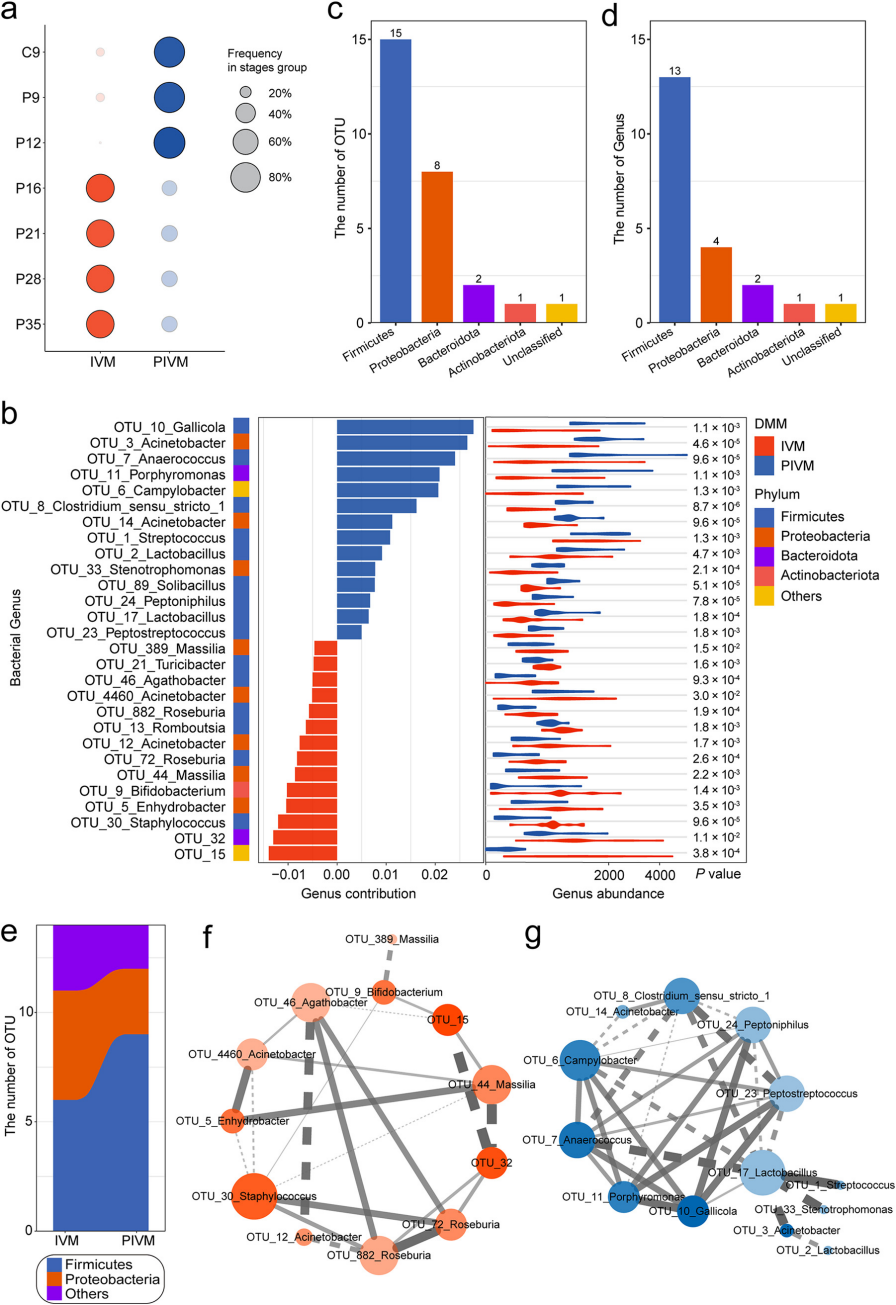
The vaginal microbiota in early pregnancy develops through stage-specific community types. (a) Distribution of samples in the two identified community types (*x* axis), clustered using Dirichlet multinomial mixtures at each pregnancy stage (*y* axis). Sizes of circles are scaled according to the OTU frequency of each community type within each stage. (b) Top 28 OTUs with more than 50% interpretation of two community types. The color bands on the left side represent the different phyla to which OTU belong. The bar plots indicate the contribution of the corresponding OTU to two community types. Statistical analysis (Wilcoxon rank-sum test) of the abundance of the 28 OTUs showing significant differences between the two community types. (c) Phylum classifications of the 28 OTUs. (d) Phylum classifications of the genera represented by the 28 OTUs. (e) Transition of *Firmicutes* and *Proteobacteria* between the two community types. The vertical axis represents the OTU number corresponding to the phylum level. (f) Cooccurrence network of community M1-associated OTUs. Pairwise correlations were calculated using SparCC. A connection represents a correlation of >0.3 and *P* value of <0.05. Solid and dashed lines, respectively, represent positive and negative correlations. The lines width and color intensity represent the correlation between two OTUs. The size of each node represents the connectivity degree with other OTUs, and the color intensity represents the contribution of corresponding OTUs to community type. (g) Cooccurrence network of community M2-associated OTUs.

Through the overall analysis, we found a dynamic change in sows’ vaginal microbiotas in early pregnancy. Next, we asked whether the vaginal microbiota of different pregnancy stages could be clustered into communities according to the stage using Dirichlet multinomial mixtures (DMM) (31). Considering sequencing artifacts and contamination, we selected 5% of the total OTUs, that is, the top 341 OTUs with the most abundance, for subsequent analysis (Fig. S4a). These OTUs contributed more than 75% of total OTU abundance, which could highly represent the function of overall microbiotas (32). We identified 2 community types that are related to pregnancy development (Fig. 2a), community types corresponding to early and later periods. According to the early pregnancy development status in pigs, we defined these types as PIVM (peri-implantation vaginal microbiota, pregnancy recognition stages before porcine embryo implantation [33]) and IVM (implantation vaginal microbiota, porcine embryo attach and implantation stages [34]).

10.1128/msystems.01192-22.4 FIG S4 Microbiota-based classification of pregnancy stages. (a) Contribution of each OTU to total OTU abundance. The dashed line represents the 341st OTU in descending order of abundance; that is, the top 5% OTUs contributed more than 75% of the total OTU abundance. (b) Contribution of each OTU to two community types. The top 28 OTUs explained more than 50% of community types. (c) NMDS analysis of the 28 OTUs in the two community types. (d) Genus classification of the 28 OTUs. Download FIG S4, TIF file, 1.7 MB. Copyright © 2023 Zang et al. 2023 Zang et al. https://creativecommons.org/licenses/by/4.0/ This content is distributed under the terms of the Creative Commons Attribution 4.0 International license.

We ranked OTUs by the interpretation degree of the pregnancy community type classification. Subsequently, the top 28 OTUs with an interpretation rate of over 50% attracted our attention for the following detailed analysis (Fig. 2b, Fig. S4b). An NMDS analysis highlights the role of these 28 OTUs in differentiating IVM (red dots) from PIVM (blue dots, Fig. S4c). The abundance of these OTUs is significantly diverse in two communities, as expected (Fig. 2b; Wilcoxon rank-sum test, *P < *0.05), suggesting that diverse communities have different functions during pregnancy. Corresponding to the number of microbes from different phyla, these OTUs that may exert certain roles belong to the top 5 phyla (Fig. 2c). Some different OTUs correspond to the common genus, indicating that these same genus microbes may be from different species (Fig. 2d, Fig. S4d) and perform individual functions in the vagina. Notably, in agreement with the abundance dynamics of *Firmicutes* and *Proteobacteria* in Fig. 1d, there are more microbes from *Firmicutes* in PIVM, while the number of *Proteobacteria* in IVM is higher (Fig. 2d). These results repeatedly demonstrate that *Firmicutes* from the vagina may assist sows to complete pregnancy recognition at the beginning of pregnancy, while *Proteobacteria* help to complete implantation through metabolic regulation (35, 36).

In addition, we found that these 28 OTUs are equally divided into two communities (Fig. 2b). To understand the interactions between microbial communities under different pregnancy states, we used network principles to express cooccurrence relationships. There is an undoubted difference between the microbial community structure related to PIVM and that related to IVM (Fig. 2f to g), and a significant SparCC (sparse correlations for compositional data) correlation between these microbes (SparCC, >0.3, *P < *0.05). For microbes associated with IVM, the taxa with the highest cooccurrence relationship, OTU_30_Staphylococcus, is positively correlated with OTU_72_*Roseburia* and OTU_882_*Roseburia*, but negatively correlated with OTU_4460_*Acinetobacter*, OTU_5_*Enhydrobacter*, and OTU_44_*Massilia* (Fig. 2f). We found that OTU_72 and OTU_882 both represent *Roseburia*, suggesting that Staphylococcus may taking part in commensalism with *Roseburia* to play shared roles. Studies have found that both Staphylococcus and *Roseburia* are related to sex hormone levels, which may affect the regulation of pregnancy-related hormones via the lower reproductive tract to achieve embryo implantation during early pregnancy (37). Of the microbes in PIVM, the most discriminative taxon, OTU_17_*Lactobacillus*, is mostly negatively correlated with other taxa such as OTU_3_*Acinetobacter*, OTU_7_*Anaerococcus*, OTU_24_*Peptoiphilus*, etc. (Fig. 2g). Notably, OTU_2_*Lactobacillus* also belongs to the genus *Lactobacillus* and is negatively correlated with OTU_3_*Acinetobacter*, but the strict criteria of the cooccurrence network resulting in a relatively positive correlation between OTU_2_*Lactobacillus* and OTU_17_*Lactobacillus* has not been shown in Fig. 2g.

For *Lactobacillus*, many studies have proved that *Lactobacillus* is ubiquitous in female vaginas, which could regulate the microenvironment in the vagina by producing lactic acid to inhibit the development of pathogenic bacteria and has an essential role in maintaining health and homeostasis (23, 38). Previous studies confirmed that the microbial community in female vaginas experience postdelivery disturbances, leading to a decrease in lactobacilli and an increase in various anaerobic bacteria, including *Peptoniphilus*, *Prevotella*, and *Anaerococcus* species (39), while a recent study found that the abundance of lactobacilli in predelivery female vaginas gradually decreases with the increase of other microbiotas (17), which is consistent with the dynamics of the vaginal microbial community in women with preterm births (13), implying the importance of these other microbes to childbirth. When microbial community changed to the community type of childbirth in advance, premature delivery followed, showing that vaginal microbiotas are closely related to pregnancy, and these microbiotas from the lower reproductive tract also could affect pregnancy development.

### Dynamic and vaginal microbiota in sows during early pregnancy.

**FIG 3:**
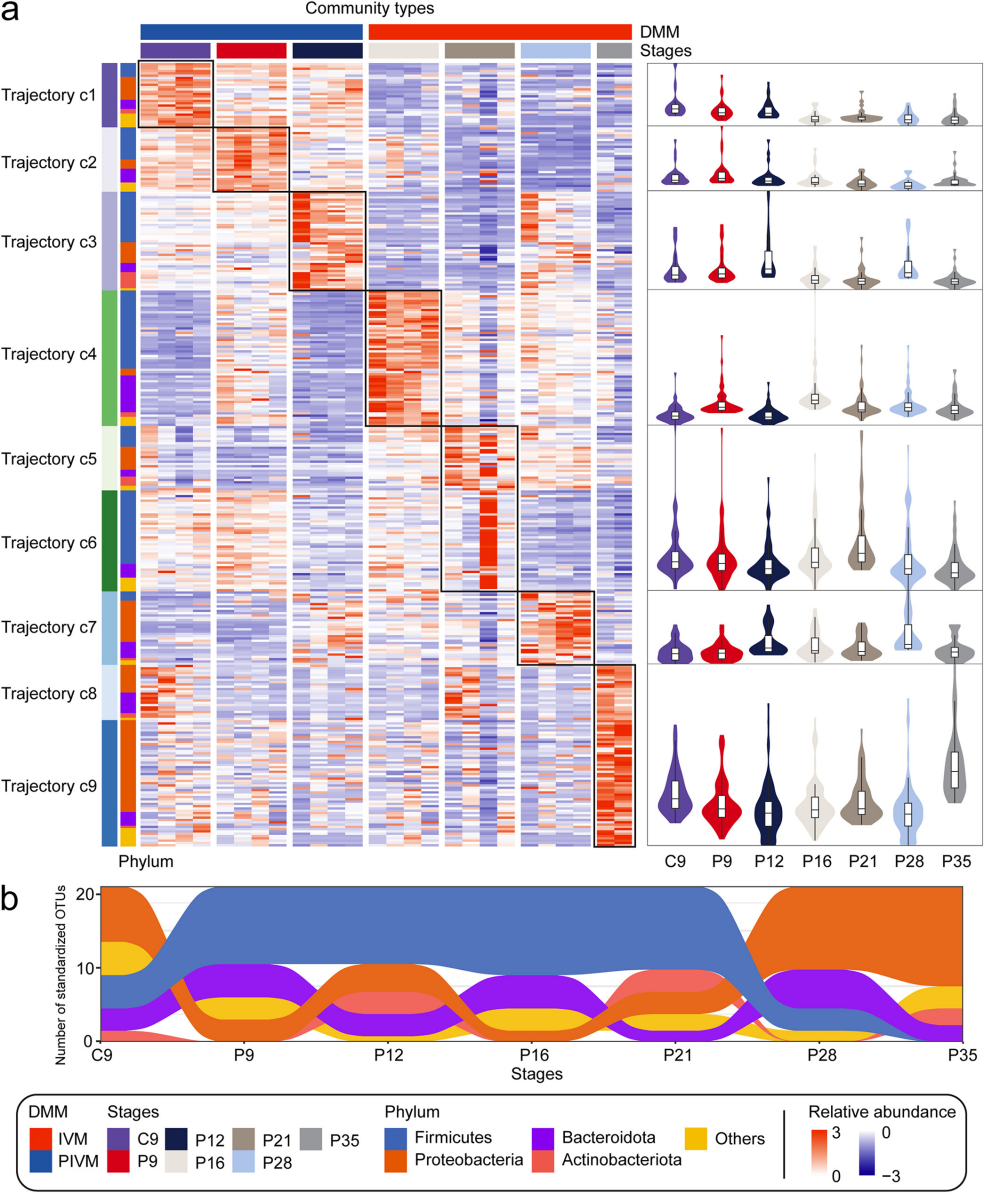
Abundance of microbial genera during the development of sow vaginal flora in early pregnancy. (a) Relative abundance heatmap of community types discriminating OTUs, sorted by the trajectories they belong to. The relative abundance of samples is sorted by stages in columns. Violin plots on the right show the relative abundance of 7 stages for high-abundance OTUs. (b) The corresponding phylum classification after standardization of the OTUs in each stage.

To further characterize the dynamics in vaginal microbiota development, we analyzed pig vagina samples in early pregnancy based on the OTU abundance using the time course of fuzzy clustering (40). The analysis identified 9 main trajectories corresponding to the high abundance of microbes at various stages in developing vaginal microbiota (Fig. 3a, Fig. S5; see Table S2 at https://zenodo.org/record/7537183).

10.1128/msystems.01192-22.5 FIG S5 Abundance of microbial genera during the development of sow vaginal flora in early pregnancy. Each cluster displays OTU sets with the same abundance variation pattern, shown with a purple trajectory line. Each line represents the abundance trend of one OTU, and yellow corresponds to OTUs with a higher membership value. Download FIG S5, TIF file, 1.1 MB. Copyright © 2023 Zang et al. 2023 Zang et al. https://creativecommons.org/licenses/by/4.0/ This content is distributed under the terms of the Creative Commons Attribution 4.0 International license.

As expected, the colonization and abundance of microbes at various stages dominate the types of pregnancy microbial communities. Specifically, microbes following trajectories c1, c2, and c3 formed the community type PIVM, while microbes following trajectories c4, c5, c6, c7, c8, and c9 formed the community type IVM (Fig. S6). These results indicate that these microbes from the lower reproductive tract may affect pregnancy development by interacting with the host. Furthermore, we found that OTU numbers do not completely match the trend of phylum abundance at different stages (Fig. 3b), suggesting that the dynamics of number and abundance of OTUs in the phylum were not consistent. For example, for *Firmicutes* or *Proteobacteria*, a higher OTU number may correspond to a lower OTU abundance, but we can still see that with the development of pregnancy, *Firmicutes* gradually decreases and *Proteobacteria* gradually increases.

### Sow vaginal epithelial transcriptomes.

**FIG 4:**
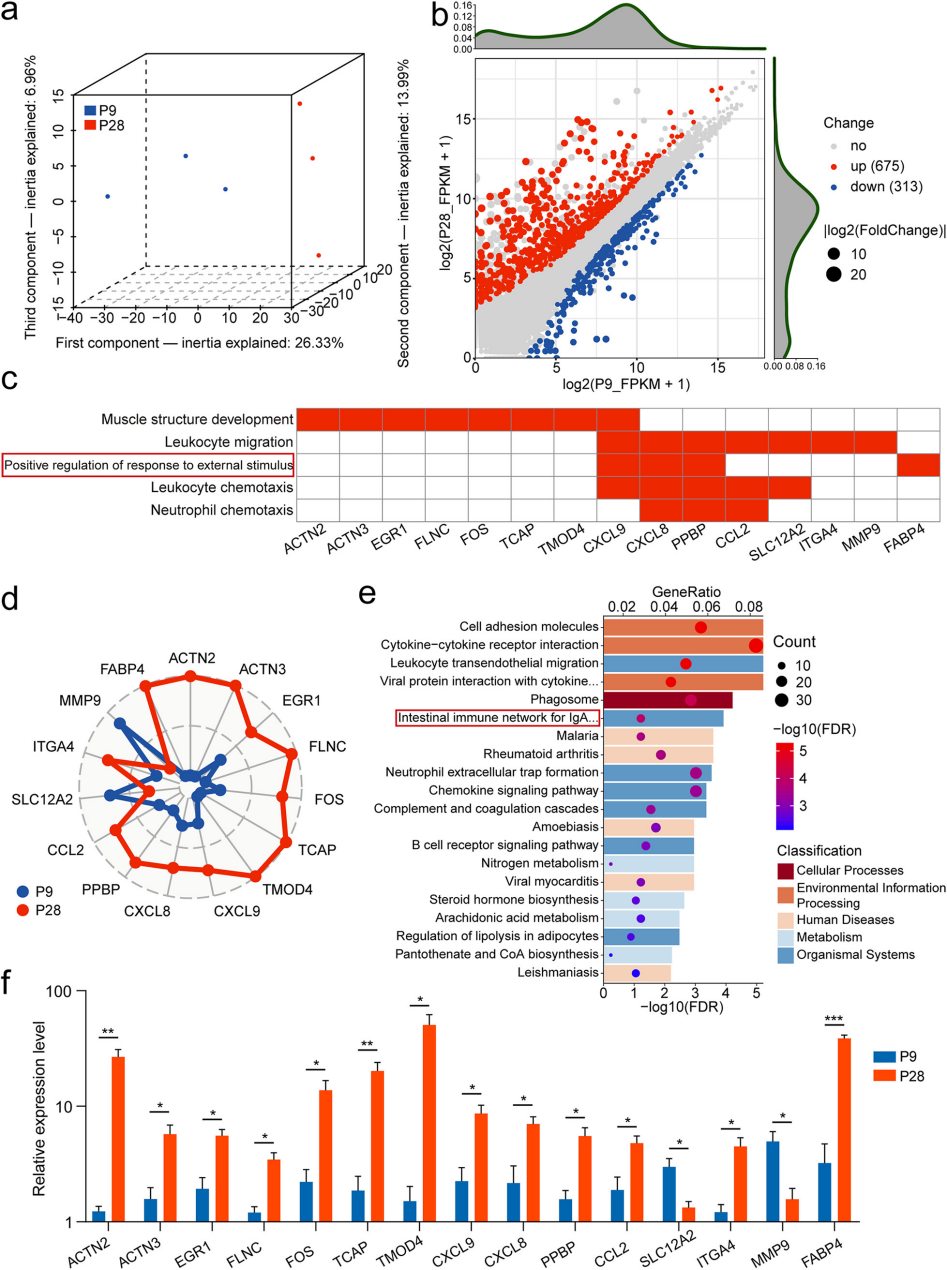
Transcriptome of the sow vaginal epithelium on days 9 and 28 of pregnancy. (a) Projection of the sample transcriptome on days 9 and 28 of pregnancy in the subspace, which is described by the three first components of the principal-component analysis (PCA). (b) DEG expression profile by scatterplot. Each point represents one gene. The red points represent upregulated genes on day 28 of pregnancy, while the blue points represent downregulated genes. (c) Top 5 biological processes annotated by DEGs and the important host regulatory genes included. (d) The radar plot shows the median normalized expression of 15 host genes in vaginal epithelial cells split by two pregnancy stages, and ticks show an increase in expression from inside to outside the circle. (e) KEGG pathway analysis of DEGs. The different colors represent the categories to which the KEGG pathways belong. The dot color corresponds to the significance of gene enrichment, and GeneRatio indicates the ratio of genes enriched to the total number of genes in the pathway. (f) Validation of the expression of genes using qRT-PCR. The relative expression level was normalized by log_10_. Data are displayed as the mean ± standard error of the mean (SEM) values (*n* = 3). The asterisks indicate statistically significant differences: *, *P < *0.5; **, *P < *0.01; ***, *P < *0.001.

To understand the interaction between vaginal microbes and the host, we considered two pregnancy communities and performed calculations for microbial community structure differences across diverse stages using Adonis (41). Finally, taking into account the degree of sample interpretation, two stages, days 9 and 28, of pregnancy with the most significant differences were selected to represent the communities PIVM and IVM, respectively, for follow-up analysis (see Table S3 at https://zenodo.org/record/7537183). We collected the additional sow vaginal epithelium on days 9 and 28 of pregnancy for transcription analysis to observe gene expression differences in the host. Corresponding to the significant changes in microbiotas, the transcriptome in the host also changed drastically. Principal-component analysis reveals significant differences between two pregnancy stages (Fig. 4a). Based on a restriction of a fold change of >2 and false-discovery rate (FDR) of <0.05, we identified a total of 988 differentially expressed genes (DEGs), 675 upregulated DEGs and 313 downregulated DEGs (Fig. 4b, Fig. S7a; see Table S4 at https://zenodo.org/record/7537183). We found that the number of upregulated DEGs is more than twice that of downregulated DEGs, showing that more genes are needed to achieve the regulation of vaginal development during pregnancy. Among the top upregulated genes, we have observed some subnovel genes without determined gene symbol which may play important functions during vaginal pregnancy (Fig. S7b). Other genes include a lot of genes involved in inflammatory response, such as IOD1, ARG1, SPIB, PAX5, MZB1, and CD79A, matrix genes (MSTN, CNFN), and a lipid metabolism gene (PLIN1). Previous studies have confirmed that pregnancy is a unique immune condition. During early pregnancy, a necessary inflammatory response occurs at the maternal-fetal interface of the upper reproductive tract to make the semialogenous fetus obtain maternal immune tolerance, thereby completing the embryo implantation (42, 43). Our results revealed that the inflammatory response that also occurs in the lower reproductive tract is not of much concern and may function together with the inflammation of the endometrial interface to promote embryo implantation. The top downregulated genes included some metabolic genes such as CYP26A1, ABAT, GNAL, and IRX2 (Fig. S7b).

10.1128/msystems.01192-22.6 FIG S6 Cooccurrence network of the trajectory OTUs. (a to g) Cooccurrence network of OTUs contained in trajectories c1, c2, c3, c4, c5 and c6, c7, and c8 and c9, respectively. Pairwise correlations were calculated using SparCC. A connection represents a correlation of >0.3 and *P* value of <0.05. Red and green lines, respectively, represent positive and negative correlations. The size of each node represents the connectivity degree with other OTUs, and the color represents the phylum to which the OTU belongs. Download FIG S6, TIF file, 2.7 MB. Copyright © 2023 Zang et al. 2023 Zang et al. https://creativecommons.org/licenses/by/4.0/ This content is distributed under the terms of the Creative Commons Attribution 4.0 International license.

10.1128/msystems.01192-22.7 FIG S7 Transcriptome of the sow vaginal epithelium on days 9 and 28 of pregnancy. (a) Hierarchical clustering heatmap of DEG expression profiles. The color scale is from −2.0 (blue, lower gene expression level) to 2.0 (red, higher gene expression level). Each row represents one gene, and each column represents one sample. (b) Top DEGs in the vaginal epithelium transcriptome. The top 25 up- and downregulated genes organized according to rank value (–log FDR × log_2_ fold change). The bar color intensity corresponds to the significance of the DEGs. (c) GO annotation terms for DEGs. The first lap represents the top 10 GO terms under distinct classification levels, and the gene number corresponds to the outer lap. The second lap represents gene numbers enriched in the specified term, and the color shade corresponds to the significance of the enrichment term. The third lap represents the ratio of the up- (red) and downregulated genes (blue). The fourth lap represents the enrichment factor of each GO term. GO, gene ontology; BP, biological process; CC, cellular component; MF, molecular function. Download FIG S7, TIF file, 0.9 MB. Copyright © 2023 Zang et al. 2023 Zang et al. https://creativecommons.org/licenses/by/4.0/ This content is distributed under the terms of the Creative Commons Attribution 4.0 International license.

Then we conducted a functional enrichment analysis of DEGs to gain insight into roles played by these genes. Consistent with the functions exhibited by the above-mentioned top genes, Gene Ontology (GO) enrichment results show that these DEGs are mainly involved in some inflammatory responses dominated by leukocytes or neutrophils and matrix structure development (Fig. S7c; see Table S5 at https://zenodo.org/record/7537183). In addition, the biological process “positive regulation of response to external stimulus” has attracted our attention. Previous studies showed that anti-sigma factors in Bacillus subtilis could connect external stimuli with gene regulation to play a role (44). Therefore, these external stimuli are likely to be derived from vaginal microbial metabolites and lead to some adaptive changes in the host, which suggests an interaction between vaginal microbes and the host. We have identified some regulatory genes through GeneWalk (Fig. S8), combined with the GO results, and we finally identified some host genes that play important roles in sows vaginal pregnancy development (Fig. 4c). We compared the expression levels of 15 host genes belonging to 5 biological processes across the two communities. Their expression patterns are significantly different and have higher transcriptional activity at 28 days of pregnancy (P28) (Fig. 4d). Among the 15 selected important host genes, structural development and inflammatory response genes accounted for half, indicating that structural development in the vagina is accompanied by vaginal inflammatory responses with pregnancy. To ensure the accuracy of our conclusions, we used quantitative PCR (qPCR) to verify the expression of these genes in two stages and obtained a consistent result (Fig. 4f). The existence of the vaginal community with a unique microbial composition indicates the differences in vaginal microenvironmental conditions during pregnancy development, which are echoed in the host differences in the vaginal ecosystem.

10.1128/msystems.01192-22.8 FIG S8 Regulator gene identification for DEGs using GeneWalk. Each dot represents a gene, and the color intensity indicates the fraction of relevant GO terms. Regulator genes were identified based on the hypothesis that these genes have high connectivity to other input and a high fraction of relevant GO annotations. Download FIG S8, TIF file, 1.3 MB. Copyright © 2023 Zang et al. 2023 Zang et al. https://creativecommons.org/licenses/by/4.0/ This content is distributed under the terms of the Creative Commons Attribution 4.0 International license.

KEGG pathway analysis shows that these DEGs were enriched in some pathways of environmental information processing, implying that these genes may produce a specific microenvironment for the host to maintain the development of the vaginal microbiota (Fig. 4e; see Table S5 at https://zenodo.org/record/7537183). Interestingly, we found that they were enriched in the pathway “intestinal immune network.” Due to the similar villi structure, some physiological changes in the vagina may be the same as those in the intestine. Unfortunately, some previous studies only compared the differences in the microbiota composition between the vagina and gut (9, 45) and did not compare the interaction between host microenvironment and microbes in the vagina or gut. These changes in physiological processes from different spatial locations may produce a specific association and copromote the host to accomplish pregnancy development.

### Integrative analysis of the vaginal microbiome and transcriptome.

**FIG 5:**
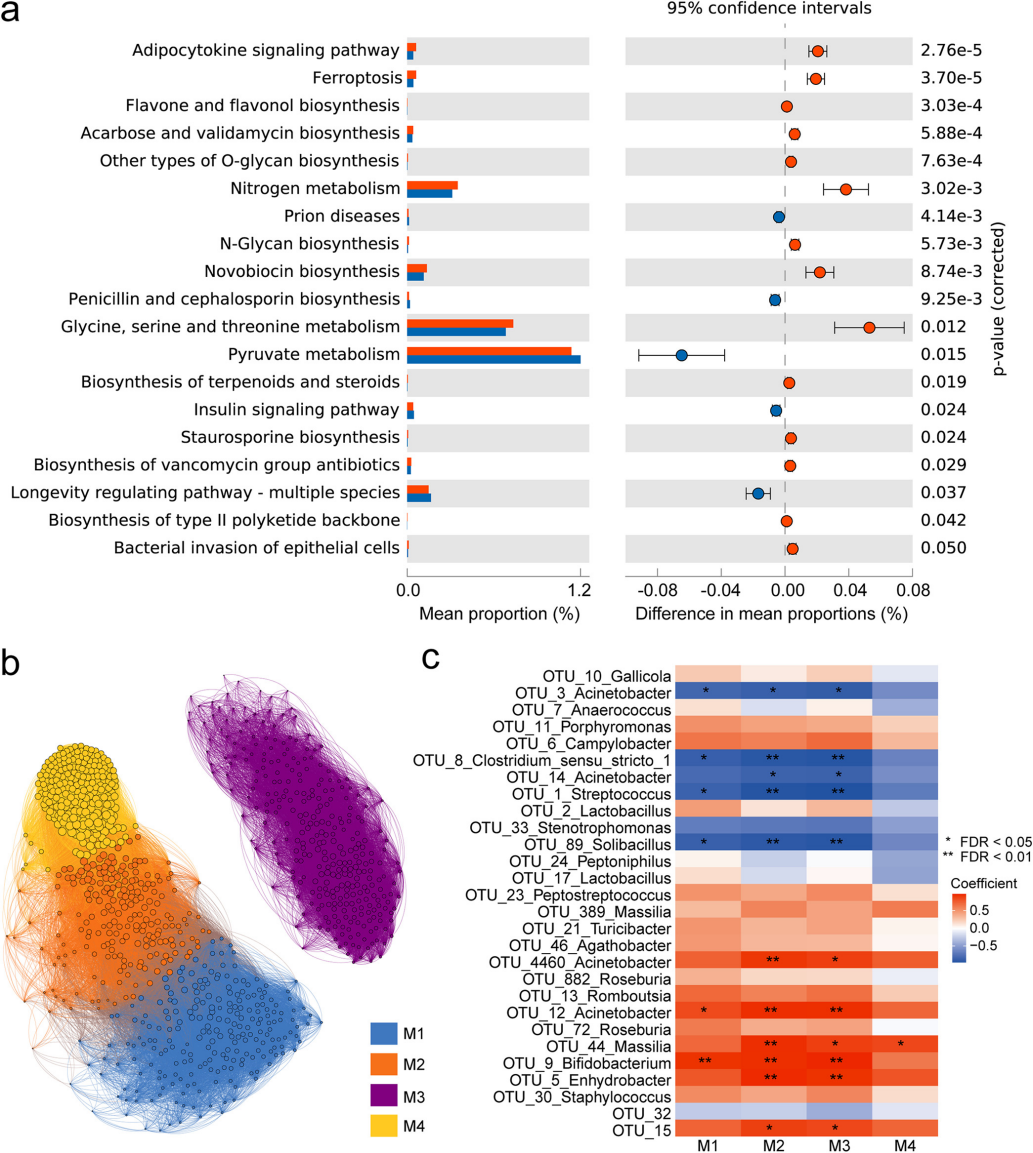
Integrative analysis of the vaginal microbiome and transcriptome. (a) The microbial pathways with significant differences between two microbial communities. The gene content of individual OTUs was inferred using the SILVA database, which was then used to predict enriched microbial pathways in the respective stages. (b) The coexpression network was constructed from DEGs between days 9 and 28 of pregnancy, resulting in a total of 82,074 pairs of connections. Subsequently, the network was partitioned into 4 modules using the Louvain method. (c) Pearson’s cocoefficient was fit for each microbial-module eigengene gene pair, and the association *P* value was corrected using the Benjamini-Hochberg method. The color of the heatmap corresponds to the association direction, and the asterisk represents the significance of association.

Taking advantage of our microbiome and transcriptome data sets, we addressed the interplay between host and microbes. First, based on the 16S rRNA gene marker, we used TaxFun2 to predict the functions of these pregnancy-specific microbes. Some metabolic and biosynthetic pathways were enriched (Fig. 5a). We found that the pathway “bacterial invasion of epithelial cells” is enriched by functions of these microbes, which corresponds to the biological process “positive regulation of response to external stimulus,” enriched by DEGs. This further proves the interaction between vaginal microbes and the host. Microbes in vagina cannot invade the vaginal epithelium, due to the mucosal barrier, but they could produce some stimulation to lead to changes in host physiological processes. To validate OTU-based predictions, we performed whole-genome sequencing (WGS) on an additional sample set. Coinertia analysis showed consistent results between two independent sequencing methods (RV, 0.66, *P < *0.05). WGS data confirmed that the expression patterns of genes related to major pathways in two pregnancy stages are consistent with the differential expression patterns of pathways enriched in two pregnancy communities (Fig. S9).

10.1128/msystems.01192-22.9 FIG S9 Validation of vaginal microbial metagenomics. WGS metagenomic abundances of genes associated with major pathways, annotated by KEGG pathway categories. Download FIG S9, TIF file, 1.4 MB. Copyright © 2023 Zang et al. 2023 Zang et al. https://creativecommons.org/licenses/by/4.0/ This content is distributed under the terms of the Creative Commons Attribution 4.0 International license.

Next, we investigated the vaginal gene expression profiles between two stages. We created a coexpression network based on Pearson correlation using the DEGs of two pregnancy stages and partitioned it into four distinct modules (Fig. 5b) (46). Consistent with the above-mentioned overall enrichment results of DEGs, the genes in these modules are also enriched in processes such as inflammatory response and matrix structure development, but the genes in each module are specifically enriched in more detail by consistent biological processes or pathways (see Table S6 at https://zenodo.org/record/7537183). For instance, module M4 was only enriched in various muscle development regulation processes, indicating that these genes from M4 collectively complete tight regulation of muscle development and differentiation. Interestingly, genes from M4 are densely clustered together in the coexpression network, implying that these genes interact closely to promote structural changes during pregnancy development.

By calculating the module eigengenes, we determined the associations between these modules and the top 28 most important microbial taxa described above. Overall, we identified 29 associations (FDR, <0.05), including the positive and negative associations (Fig. 5c). Notably, we found that the microbes associated with M2 are also associated with M3, but not all of them are associated with M1, which suggests that the actual physiological process between M2 and M3 may be closer. However, for the DEG coexpression network, the distance between M2 and M3 is markedly greater than that between M1 and M2. For the biological processes of module enrichment, M1 and M2 enriched some immune and inflammatory responses, while M3 enriched the vasculature development (Fig. S10). These seemingly conflicting results may be caused by some other potential regulatory mechanisms. Most importantly, only one positive association was identified between M4 and OTU_44_*Massilia*. Previous studies have found that the gut microbiota and its metabolites both could affect muscle development (47, 48). M4 is strongly related to muscle development and differentiation (Fig. S10g), indicating that OTU_44_ *Massilia* may well play an important role in promoting vaginal muscle differentiation and development. In summary, our results demonstrate that the microbes in the vagina could interact with the host to promote pregnancy development. The communication between host and microbiotas depends on the defined gene subset, and the interaction is presumably bidirectional, with the microbiotas influencing host gene expression, which in turn forms a habitat for specific microbes.

10.1128/msystems.01192-22.10 FIG S10 Functional analysis of the microbial-related modules using clusterProfiler. (a, c, e, and g) GO annotation terms for genes contained in modules M1, M2, M3, and M4, respectively. Blue represents the biological process in the GO terms, orange represents cellular component, and purple represents molecular function. (b, d, f, and h) KEGG pathway analysis of genes in modules M1, M2, M3, and M4, respectively. The dot color corresponds to the significance of gene enrichment, and GeneRatio indicates the ratio of genes enriched to the total number of genes in the pathway. Download FIG S10, TIF file, 1.1 MB. Copyright © 2023 Zang et al. 2023 Zang et al. https://creativecommons.org/licenses/by/4.0/ This content is distributed under the terms of the Creative Commons Attribution 4.0 International license.

**FIG 6:**
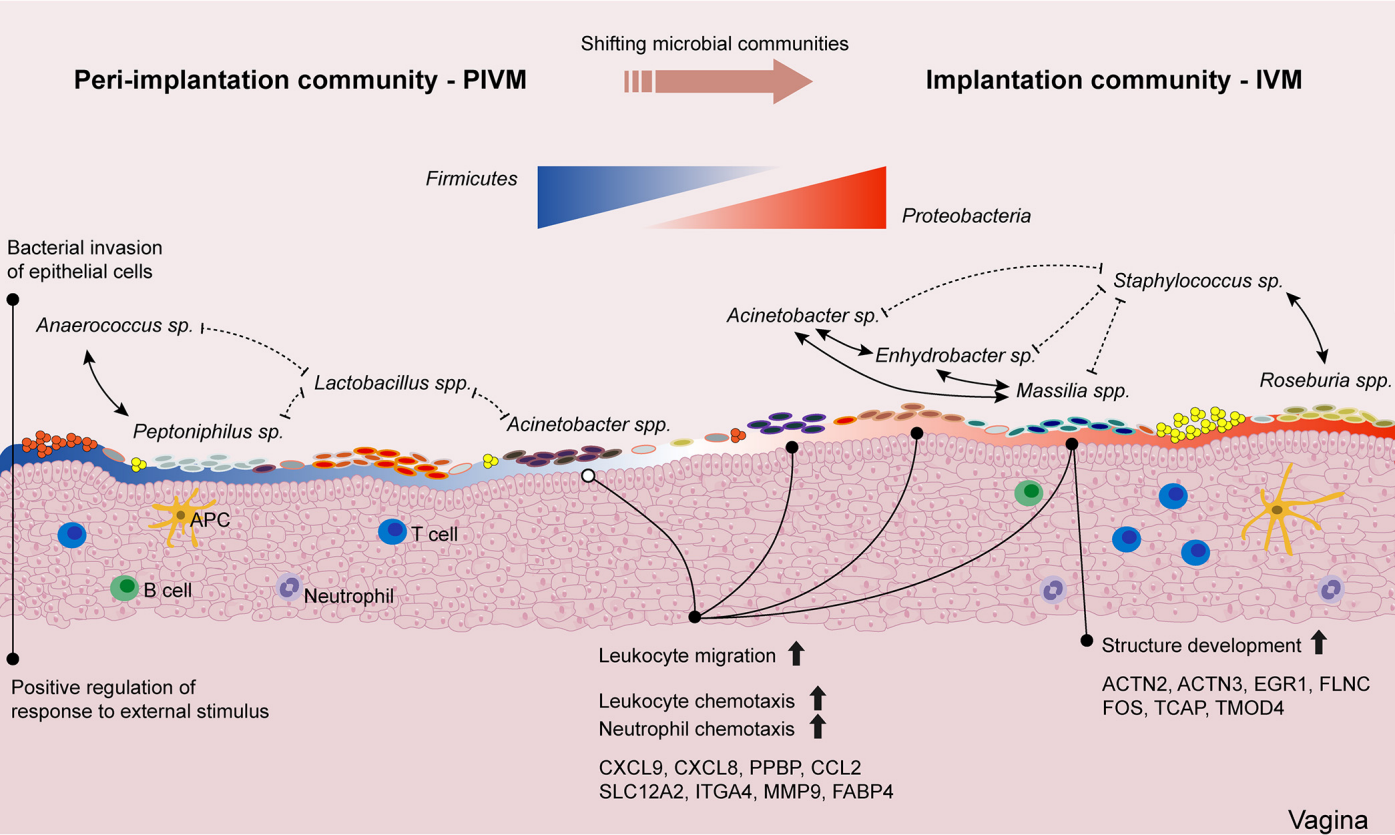
Interaction between host and microbes in vagina. The vaginal microbial community of sows changes dynamically with pregnancy development. PIVM is characterized by a relatively high abundance of *Firmicutes*, while IVM shows the high abundance of *Proteobacteria*, which are related to multiple species, and no single species dominates. Microbial invasion stimulates epithelial cells, and vaginal epithelium produces positive regulation in response to stimulation. Dotted lines represent antagonism; solid lines represent synergy.

**Conclusion.** This study determined an antagonistic relationship between *Proteobacteria* and *Firmicutes* by describing the dynamic development of vaginal microbes in sows during early pregnancy. The further detailed analysis identified two pregnancy-specific microbial communities, highlighting the importance of vaginal microbes interacting with the host to promote pregnancy development (Fig. 6). Furthermore, these data sets provide a reference for the microbial-host interaction in the vagina during human female reproduction, aiming to establish a homeostatic relationship between microbes and humans for medical intervention. Unfortunately, due to experimental method limitations, the number of samples in the overall cohort is still small. Although an additional WGS method was used to verify the data, the sample population will need to be expanded in the future to explore the complete genetic diversity of microorganisms.

## MATERIALS AND METHODS

### Experimental design and sample collection.

This study was approved by the Ethics Committees of the Laboratory Animal Center of South China Agricultural University (permit number SYXK-2019-0136).

A total of 28 healthy and disease-free Tibetan sows (parity 2) with similar physiological conditions that had been artificially fed and bred for multiple generations and were raised on a farm in Guangdong, China. Subsequently, these sows were randomly divided into 7 groups with 4 sows in each group, including the cyclic group (*n* = 4) and 6 different pregnancy groups (*n* = 24). The sows were checked for estrus twice a day. After estrus, sows in the pregnancy group were administered standard doses of purebred boar semen for artificial insemination, while sows belonging to the cyclic group were given dead semen from the same boar. Generally, vaginal swabs from the same individual were used to study the ecological succession of developing vaginal microbiotas (17). Considering the relatively close sampling period of this study, to avoid the effects of vaginal swabs collected during the previous pregnancy stage for vaginal microbial communities at other subsequent stages and additional factors caused by environmental microbes, we decided to slaughter these sows to collect vaginas for this study. The sows’ intact uteruses were collected at a local slaughterhouse on day 9 of the estrous cycle (C9) and days 9, 12, 16, 21, 28, and 35 of pregnancy (P9, P12, P16, P21, P28, and P35) and quickly transferred to a sterile fume hood in an ice box. We removed the partial vagina exposed to the air using sterile scissors and then rubbed the vagina 5 times with a swab. The swabs were quick-frozen in liquid nitrogen and stored at −80°C to preserve the vaginal microbiota for DNA extraction. Unfortunately, only two samples were finally collected on P35 for additional reasons.

Vaginal swabs were collected from six additional sows on days 9 and 28 using the procedure described above for metagenomic shotgun analysis. The sows’ vaginas were then opened longitudinally, and a cell scraper was used to collect the vaginal epithelium, which was immediately immersed in liquid nitrogen and then transferred to a 80°C freezer for subsequent RNA extraction. Final analysis included 32 vaginal swabs and 6 vaginal epithelial samples integrating the microbiome with the transcriptome.

### 16S rRNA gene sequencing and analyses.

DNA was extracted from vaginal swabs using the cetyltrimethylammonium bromide (CTAB) method as previously described (49). The DNA concentration and purity were monitored on 1% agarose gels, and then DNA samples were diluted to 1 ng/μL using sterile water. PCR amplification was conducted using the primer pairs 341F (forward primer, 5′-CCTAYGGGRBGCASCAG-3′) and 806R (reverse primer, 5′-GGACTACNNGGGTATCTAAT-3′) with the barcode targeting the variable V3 to V4 regions of the 16S rRNA gene. All PCRs were carried out with 15 μL of Phusion high-fidelity PCR master mix (New England Biolabs), 2 μM forward and reverse primers, and approximately 10 ng of template DNA. The following thermal cycle protocol was used: 98°C for 1 min, 30 cycles of 98°C for 10 s, 50°C for 30 s, and 72°C for 30 s, and finally, hold at 72°C for 5 min. Each PCR was run in triplicate. The same volume of 1× loading buffer (containing SYBR green) was mixed with PCR products, and electrophoresis detection on a 2% agarose gel was performed. PCR products were mixed in an equidensity ratio, and the mixed PCR products were purified using a gel extraction kit (Qiagen, Germany). Subsequently, sequencing libraries were generated using a TruSeq DNA PCR-free sample preparation kit (Illumina, USA) following the manufacturer’s instructions, and index codes were added. A Qubit 2.0 fluorometer (Thermo Scientific, California, USA) and Bioanalyzer 2100 system (Agilent Technologies, Santa Clara, CA) were used to evaluate the library quality, the library was sequenced on an Illumina NovaSeq 6,000 platform, and 250-bp paired-end reads were generated.

Raw reads were trimmed based on their unique barcode and then were merged using FLASH (v.1.2.7) (50). To exclude potential sequencing errors, merged sequences were filtered using QIIME (v.1.9.1) (24) for quality control, and chimera sequences were identified with VSEARCH (v.2.9.0) (51). Subsequently, sequences were clustered into operational taxonomic units (OTUs) at a 97% identity threshold using UPARSE (v.7.0.1001) (52). Using Mothur algorithm (v.1.33.3) to align the SILVA database (v.138, https://www.arb-silva.de), we obtained the taxonomic information of these OTUs. To correct for differences in sequencing depth, samples were subsampled to the sample number of reads. In the analysis of relative abundance on diverse level counts, the counts were scaled counts to the total sum of counts, and values given as relative abundance sums up to 1.

Alpha diversity was calculated using QIIME to analyze the complexity of species diversity, including Good’s coverage and Shannon and Chao1 indexes. NMDS analysis was performed using the vegan R package (v.2.5-7) (53). Differences in abundance were based on counts normalized to the total sum in each sample. The overall difference was tested using the Kruskal-Wallis H test during seven stages (*P < *0.05), while the significant dynamics across these 7 stages was determined using the Jonckheere-Terpstra test (*P < *0.05).

To preserve the study power, only the top 5% of OTUs accumulating the total 75% abundance of the overall OTUs were used for detailed analysis. Sow vaginal microbiota community types were determined by clustering rarefied counts using the Dirichlet multinomial mixtures model (31). The number of community types was selected by choosing the number of components that gave the minimal Laplace approximation to the negative log model evidence (see Fig. S11 at https://zenodo.org/record/7537183). Samples were passed to community type according to their maximum posterior probability. Differences between two communities were tested using the Wilcoxon rank-sum test (*P < *0.05). Genus trajectories were calculated by soft clustering using the Mfuzz R package (v.2.42.0) (40). The alluvial diagrams illustrate the trajectory of microbial phylum proportion in diverse pregnancy stages over time using the alluvial R package (v.0.12.2) (54).

To understand the interactions between microbes, we constructed the microbial cooccurrence networks. We employed SparCC on the normalized OTU abundance, which calculates a modified correlation coefficient designed specifically for evaluating the correlative relationships between taxa in microbiome studies (55). The statistical significance of correlations was evaluated based on an empirical null distribution determined with 100 bootstrap iterations (*P < *0.05; SparCC, >0.3). Network visualizations were generated in Cytoscape (v.3.7.2) (56) and Gephi (v.0.9.2) (57).

We used Tax4Fun2 (v.1.1.5) to perform metagenomic inference of the 16S rRNA gene sequence (58). Briefly, the gene content of individual OTUs was inferred using the SILVA database, and KEGG Orthologs were extracted on multiple hierarchical levels. To test the significant difference in functional category abundances between two microbial communities, we used the Welch’s *t* test implementation of STAMP (v.2.1.3) (59). The *P* values were adjusted for multiple testing using the Sidak method (60).

### Metagenomics shotgun sequencing and analyses.

The DNA used for shotgun sequencing libraries was extracted and prepared as described above. A total amount of 1 μg DNA per sample was used as the input material for DNA sample preparations. Sequencing libraries were generated using the NEBNext Ultra DNA library prep kit for Illumina (New England BioLabs, Ipswich, MA) following the manufacturer’s recommendations and sequenced on an Illumina NovaSeq 6,000 platform to generate the 150-bp paired-end reads. Raw reads were quality controlled using Readfq (v.8), and Bowtie2 (v.2.2.4) was used to remove the sequences from the pigs (61, 62). Then clean reads were assembled using SOAPdenovo (v.2.04) (63, 64). MetaGeneMark (v.2.10) (65, 66) was used for ORF prediction, and CD-HIT (v.4.5.8) (67) was adopted to redundancy. The unigene sequences were obtained by clustering of 95% identity and 90% coverage. Meanwhile, Bowtie2 was used to calculate the unigene abundance information. The function of Unigenes was predicted using DIAMOND (v.0.9.9.110) through KEGG database (68). Correlation to whole-genome sequencing was made on metagenome inference from 16S rRNA gene sequences using the ade4 function in the R package to perform coinertia analysis and estimate the *P* value.

### RNA sequencing and analyses.

Total RNA of vaginal epitheliums was extracted using TRIzol reagent (Invitrogen, Carlsbad, CA) following the manufacturer’s instructions. The RNA integrity and quantity were assessed using the RNA Nano 6,000 assay kit in the Bioanalyzer 2100 system (Agilent Technologies, Santa Clara, CA). A total amount of 1 μg RNA per sample was used as the input material for RNA sample preparations. Subsequently, following to the manufacturer’s protocols, the NEBNext Ultra RNA library prep kit for Illumina (New England BioLabs, Ipswich, MA) was used to prepare sequencing libraries with index labels. The clustering of the index-coded samples was performed on a cBot cluster-generation system using the TruSeq PE cluster kit v3-cBot-HS (Illumina, San Diego, CA) following the manufacturer’s instructions. After cluster generation, the libraries were sequenced on an Illumina NovaSeq 6,000 platform, and 150-bp paired-end reads were generated.

The raw reads were trimmed by removing adapter sequences, reads with unknown bases, and low-quality reads with more than 50% nucleotides with a Qphred score of ≤20. All the downstream analyses were based on the clean data with high quality. Then, the clean reads for each sample were aligned to the reference genome (Sscrofa11.1, http://asia.ensembl.org/Sus_scrofa/Info/Index) using the software HISAT2 (v.2.0.5) (69), and the mapped reads were transferred to StringTie (v.1.3.3b) (70) for transcript assembly. The read numbers mapped to each gene were counted using featureCounts (v.1.5.0-p3) (71). The fragments per kilobase per million (FPKM) of each gene was calculated for gene expression analysis based on the gene length and reads count mapped to this gene.

Differential expression analysis was performed between two stages using the DESeq2 R package (v.1.20.0) (72). DEGs were determined by an FDR value (*P* value adjusted by the Benjamini-Hochberg method) of <0.05 and a log_2_ fold change of >1. GO enrichment and KEGG pathway analysis were implemented with the clusterProfiler R package (v.4.0.5) (73), and the regulator genes of DEGs were identified using GeneWalk (v.1.5.3) (74).

### Quantitative real-time PCR.

The total RNA isolated from the vaginal epithelium was subjected to quantitative real-time PCR (qRT-PCR). Briefly, we synthesized cDNA using the Evo M-MLV RT kit with gDNA Clean for qPCR II (Accurate Biology, China). We then performed qRT-PCR employing the SYBR Green premix Pro *Taq* HS qPCR kit (Accurate Biology, China) following the manufacturer’s protocol. We adopted the following qRT-PCR protocol: 95°C for 10 min, 40 cycles of 95°C for 15 s, 60°C for 30 s, and 72°C for 20 s. Primers were determined using NCBI Primer-BLAST, and all reactions were repeated in triplicate (see Table S7 at https://zenodo.org/record/7537183). The relative expression of genes was calculated via the 2^−ΔΔ^*^CT^* method and normalized using β-actin (ACTB).

### Identification of host-microbial interactions.

We used the DEGs identified above to identify the interaction between host and microbes. These DEGs for integration were calculated via the pairwise Pearson correlations to construct a coexpression network. By determining a threshold of a *P* value of <0.05 and extremely correlated coefficient *r* of >0.9, we further selected the main components of the network. Subsequently, the Louvain method for community detection was employed to partition the network into gene modules showing similar expression profiles (75). The coexpression network was visualized in Gephi.

For each module identified through network community detection, dimensionality reduction was performed to determine the putative module eigengene by calculating the first principal component of module gene expression. Subsequently, the Pearson correlation coefficient was also used to test the association between microbes and module eigengenes. The *P* values were corrected using the Benjamini-Hochberg method, and the association of the microbe-module with an FDR of <0.05 was considered significant. Finally, for the modules with significant microbial associations, we used clusterProfiler to perform functional enrichment analysis to evaluate the effect of these microbes on the host.

### Data availability.

The sequencing data of the 16S rRNA gene sequence are available in the NCBI Sequence Read Archive (SRA) under accession number PRJNA788181. The metagenomic sequence data are available in the NCBI SRA under accession number PRJNA788183. Transcriptome sequencing data are available via the NCBI SRA under accession number PRJNA788184.

## References

[1] Lunney JK. 2007. Advances in swine biomedical model genomics. Int J Biol Sci 3:179–184. doi:10.7150/ijbs.3.179. 17384736 PMC1802015

[2] Schook L, Beattie C, Beever J, Donovan S, Jamison R, Zuckermann F, Niemi S, Rothschild M, Rutherford M, Smith D. 2005. Swine in biomedical research: creating the building blocks of animal models. Anim Biotechnol 16:183–190. doi:10.1080/10495390500265034. 16342425

[3] Ross JW, Ashworth MD, Stein DR, Couture OP, Tuggle CK, Geisert RD. 2009. Identification of differential gene expression during porcine conceptus rapid trophoblastic elongation and attachment to uterine luminal epithelium. Physiol Genomics 36:140–148. doi:10.1152/physiolgenomics.00022.2008. 19033546

[4] Stroband H, Van der Lende T. 1990. Embryonic and uterine development during early pregnancy in pigs. J Reprod Fertil Suppl 40:261–277. 2192043

[5] Waclawik A, Kaczmarek MM, Blitek A, Kaczynski P, Ziecik AJ. 2017. Embryo-maternal dialogue during pregnancy establishment and implantation in the pig. Mol Reprod Dev 84:842–855. doi:10.1002/mrd.22835. 28628266

[6] Wang Y, Xue S, Liu X, Liu H, Hu T, Qiu X, Zhang J, Lei M. 2016. Analyses of long non-coding RNA and mRNA profiling using RNA sequencing during the pre-implantation phases in pig endometrium. Sci Rep 6:20238. doi:10.1038/srep20238. 26822553 PMC4731748

[7] Hu Q, Zang X, Ding Y, Gu T, Shi J, Li Z, Cai G, Liu D, Wu Z, Hong L. 2022. Porcine uterine luminal fluid-derived extracellular vesicles improve conceptus-endometrial interaction during implantation. Theriogenology 178:8–17. doi:10.1016/j.theriogenology.2021.10.021. 34735978

[8] Chen C, Song X, Wei W, Zhong H, Dai J, Lan Z, Li F, Yu X, Feng Q, Wang Z, Xie H, Chen X, Zeng C, Wen B, Zeng L, Du H, Tang H, Xu C, Xia Y, Xia H, Yang H, Wang J, Wang J, Madsen L, Brix S, Kristiansen K, Xu X, Li J, Wu R, Jia H. 2017. The microbiota continuum along the female reproductive tract and its relation to uterine-related diseases. Nat Commun 8:875. doi:10.1038/s41467-017-00901-0. 29042534 PMC5645390

[9] Nugeyre M-T, Tchitchek N, Adapen C, Cannou C, Contreras V, Benjelloun F, Ravel J, Le Grand R, Marlin R, Menu E. 2019. Dynamics of vaginal and rectal microbiota over several menstrual cycles in female cynomolgus macaques. Front Cell Infect Microbiol 9:188. doi:10.3389/fcimb.2019.00188. 31249812 PMC6582644

[10] Matějková T, Hájková P, Stopková R, Stanko M, Martin J-F, Kreisinger J, Stopka P. 2020. Oral and vaginal microbiota in selected field mice of the genus Apodemus: a wild population study. Sci Rep 10:13246. doi:10.1038/s41598-020-70249-x. 32764739 PMC7413396

[11] Chen T, Xia C, Hu H, Wang H, Tan B, Tian P, Zhao X, Wang L, Han Y, Deng K-Y, Wei H, Xin H-B. 2021. Dysbiosis of the rat vagina is efficiently rescued by vaginal microbiota transplantation or probiotic combination. Int J Antimicrob Agents 57:106277. doi:10.1016/j.ijantimicag.2021.106277. 33434677

[12] Ravel J, Gajer P, Abdo Z, Schneider GM, Koenig SSK, McCulle SL, Karlebach S, Gorle R, Russell J, Tacket CO, Brotman RM, Davis CC, Ault K, Peralta L, Forney LJ. 2011. Vaginal microbiome of reproductive-age women. Proc Natl Acad Sci USA 108:4680–4687. doi:10.1073/pnas.1002611107. 20534435 PMC3063603

[13] Fettweis JM, Brooks JP, Serrano MG, Sheth NU, Girerd PH, Edwards DJ, Strauss IJ, Jefferson KK, Buck GA, The Vaginal Microbiome Consortium. 2014. Differences in vaginal microbiome in African American women versus women of European ancestry. Microbiology (Reading) 160:2272–2282. doi:10.1099/mic.0.081034-0. 25073854 PMC4178329

[14] Chen YE, Fischbach MA, Belkaid Y. 2018. Skin microbiota: host interactions. Nature 553:427–436. doi:10.1038/nature25177. 29364286 PMC6075667

[15] Serrano MG, Parikh HI, Brooks JP, Edwards DJ, Arodz TJ, Edupuganti L, Huang B, Girerd PH, Bokhari YA, Bradley SP, Brooks JL, Dickinson MR, Drake JI, Duckworth RA, Fong SS, Glascock AL, Jean S, Jimenez NR, Khoury J, Koparde VN, Lara AM, Lee V, Matveyev AV, Milton SH, Mistry SD, Rozycki SK, Sheth NU, Smirnova E, Vivadelli SC, Wijesooriya NR, Xu J, Xu P, Chaffin DO, Sexton AL, Gravett MG, Rubens CE, Hendricks-Muñoz KD, Jefferson KK, Strauss JF, Fettweis JM, Buck GA. 2019. Racioethnic diversity in the dynamics of the vaginal microbiome during pregnancy. Nat Med 25:1001–1011. doi:10.1038/s41591-019-0465-8. 31142850 PMC6746180

[16] Romero R, Hassan SS, Gajer P, Tarca AL, Fadrosh DW, Nikita L, Galuppi M, Lamont RF, Chaemsaithong P, Miranda J, Chaiworapongsa T, Ravel J. 2014. The composition and stability of the vaginal microbiota of normal pregnant women is different from that of non-pregnant women. Microbiome 2:10. doi:10.1186/2049-2618-2-4. 24735933 PMC4022389

[17] Rasmussen MA, Thorsen J, Dominguez-Bello MG, Blaser MJ, Mortensen MS, Brejnrod AD, Shah SA, Hjelmsø MH, Lehtimäki J, Trivedi U, Bisgaard H, Sørensen SJ, Stokholm J. 2020. Ecological succession in the vaginal microbiota during pregnancy and birth. ISME J 14:2325–2335. doi:10.1038/s41396-020-0686-3. 32488167 PMC7609337

[18] Gorodeski GI, Hopfer U, Liu CC, Margles E. 2005. Estrogen acidifies vaginal pH by up-regulation of proton secretion via the apical membrane of vaginal-ectocervical epithelial cells. Endocrinology 146:816–824. doi:10.1210/en.2004-1153. 15498880 PMC2398721

[19] Witkin SS, Linhares IM. 2017. Why do lactobacilli dominate the human vaginal microbiota? BJOG 124:606–611. doi:10.1111/1471-0528.14390. 28224747

[20] Anahtar MN, Gootenberg DB, Mitchell CM, Kwon DS. 2018. Cervicovaginal microbiota and reproductive health: the virtue of simplicity. Cell Host Microbe 23:159–168. doi:10.1016/j.chom.2018.01.013. 29447695

[21] Ziklo N, Vidgen ME, Taing K, Huston WM, Timms P. 2018. Dysbiosis of the vaginal microbiota and higher vaginal kynurenine/tryptophan ratio reveals an association with Chlamydia trachomatis genital infections. Front Cell Infect Microbiol 8:1. doi:10.3389/fcimb.2018.00001. 29404279 PMC5778109

[22] Shannon B, Gajer P, Yi TJ, Ma B, Humphrys MS, Thomas-Pavanel J, Chieza L, Janakiram P, Saunders M, Tharao W, Huibner S, Shahabi K, Ravel J, Kaul R. 2017. Distinct effects of the cervicovaginal microbiota and herpes simplex type 2 infection on female genital tract immunology. J Infect Dis 215:1366–1375. doi:10.1093/infdis/jix088. 28201724 PMC5451606

[23] Chee WJY, Chew SY, Than LTL. 2020. Vaginal microbiota and the potential of Lactobacillus derivatives in maintaining vaginal health. Microb Cell Fact 19:1–24. doi:10.1186/s12934-020-01464-4. 33160356 PMC7648308

[24] Caporaso JG, Kuczynski J, Stombaugh J, Bittinger K, Bushman FD, Costello EK, Fierer N, Peña AG, Goodrich JK, Gordon JI, Huttley GA, Kelley ST, Knights D, Koenig JE, Ley RE, Lozupone CA, McDonald D, Muegge BD, Pirrung M, Reeder J, Sevinsky JR, Turnbaugh PJ, Walters WA, Widmann J, Yatsunenko T, Zaneveld J, Knight R. 2010. QIIME allows analysis of high-throughput community sequencing data. Nat Methods 7:335–336. doi:10.1038/nmeth.f.303. 20383131 PMC3156573

[25] Roswall J, Olsson LM, Kovatcheva-Datchary P, Nilsson S, Tremaroli V, Simon M-C, Kiilerich P, Akrami R, Krämer M, Uhlén M, Gummesson A, Kristiansen K, Dahlgren J, Bäckhed F. 2021. Developmental trajectory of the healthy human gut microbiota during the first 5 years of life. Cell Host Microbe 29:765–776.e3. doi:10.1016/j.chom.2021.02.021. 33794185

[26] Luque AT, Fontana C, Pasteris SE, Bassi D, Cocconcelli PS, Otero MC. 2021. Vaginal bacterial diversity from healthy gilts and pregnant sows subjected to natural mating or artificial insemination. Res Vet Sci 140:26–37. doi:10.1016/j.rvsc.2021.07.023. 34391059

[27] Mendes R, Kruijt M, de Bruijn I, Dekkers E, van der Voort M, Schneider JHM, Piceno YM, DeSantis TZ, Andersen GL, Bakker PAHM, Raaijmakers JM. 2011. Deciphering the rhizosphere microbiome for disease-suppressive bacteria. Science 332:1097–1100. doi:10.1126/science.1203980. 21551032

[28] Rizzatti G, Lopetuso L, Gibiino G, Binda C, Gasbarrini A. 2017. Proteobacteria: a common factor in human diseases. BioMed Res Int 2017:9351507. doi:10.1155/2017/9351507. 29230419 PMC5688358

[29] Pammi M, Cope J, Tarr PI, Warner BB, Morrow AL, Mai V, Gregory KE, Kroll JS, McMurtry V, Ferris MJ, Engstrand L, Lilja HE, Hollister EB, Versalovic J, Neu J. 2017. Intestinal dysbiosis in preterm infants preceding necrotizing enterocolitis: a systematic review and meta-analysis. Microbiome 5:31. doi:10.1186/s40168-017-0248-8. 28274256 PMC5343300

[30] Moossavi S, Sepehri S, Robertson B, Bode L, Goruk S, Field CJ, Lix LM, de Souza RJ, Becker AB, Mandhane PJ, Turvey SE, Subbarao P, Moraes TJ, Lefebvre DL, Sears MR, Khafipour E, Azad MB. 2019. Composition and variation of the human milk microbiota are influenced by maternal and early-life factors. Cell Host Microbe 25:324–335.e4. doi:10.1016/j.chom.2019.01.011. 30763539

[31] Holmes I, Harris K, Quince C. 2012. Dirichlet multinomial mixtures: generative models for microbial metagenomics. PLoS One 7:e30126. doi:10.1371/journal.pone.0030126. 22319561 PMC3272020

[32] Ling F, Hwang C, LeChevallier MW, Andersen GL, Liu W-T. 2016. Core-satellite populations and seasonality of water meter biofilms in a metropolitan drinking water distribution system. ISME J 10:582–595. doi:10.1038/ismej.2015.136. 26251872 PMC4817684

[33] Blomberg LA, Zuelke KA. 2004. Serial analysis of gene expression (SAGE) during porcine embryo development. Reprod Fertil Dev 16:87–92. doi:10.1071/RD03081. 14972106

[34] Croy BA, Waterfield A, Wood W, King GJ. 1988. Normal murine and porcine embryos recruit NK cells to the uterus. Cellular immunology 115:471–480. doi:10.1016/0008-8749(88)90199-2. 3409331

[35] Poncin K, Gillet S, De Bolle X. 2018. Learning from the master: targets and functions of the CtrA response regulator in Brucella abortus and other alpha-proteobacteria. FEMS Microbiol Rev 42:500–513. doi:10.1093/femsre/fuy019. 29733367

[36] Leyn SA, Suvorova IA, Kazakov AE, Ravcheev DA, Stepanova VV, Novichkov PS, Rodionov DA. 2016. Comparative genomics and evolution of transcriptional regulons in Proteobacteria. Microb Genom 2:e000061. doi:10.1099/mgen.0.000061. 28348857 PMC5343134

[37] He Y, Wang Q, Li X, Wang G, Zhao J, Zhang H, Chen W. 2020. Lactic acid bacteria alleviate polycystic ovarian syndrome by regulating sex hormone related gut microbiota. Food Funct 11:5192–5204. doi:10.1039/C9FO02554E. 32441726

[38] Łaniewski P, Ilhan ZE, Herbst-Kralovetz MM. 2020. The microbiome and gynaecological cancer development, prevention and therapy. Nat Rev Urol 17:232–250. doi:10.1038/s41585-020-0286-z. 32071434 PMC9977514

[39] DiGiulio DB, Callahan BJ, McMurdie PJ, Costello EK, Lyell DJ, Robaczewska A, Sun CL, Goltsman DS, Wong RJ, Shaw G, Stevenson DK, Holmes SP, Relman DA. 2015. Temporal and spatial variation of the human microbiota during pregnancy. Proc Natl Acad Sci USA 112:11060–11065. doi:10.1073/pnas.1502875112. 26283357 PMC4568272

[40] Kumar L, Futschik ME. 2007. Mfuzz: a software package for soft clustering of microarray data. Bioinformation 2:5–7. doi:10.6026/97320630002005. 18084642 PMC2139991

[41] Stat M, Pochon X, Franklin EC, Bruno JF, Casey KS, Selig ER, Gates RD. 2013. The distribution of the thermally tolerant symbiont lineage (Symbiodinium clade D) in corals from Hawaii: correlations with host and the history of ocean thermal stress. Ecol Evol 3:1317–1329. doi:10.1002/ece3.556. 23762518 PMC3678486

[42] Erlebacher A. 2013. Immunology of the maternal-fetal interface. Annu Rev Immunol 31:387–411. doi:10.1146/annurev-immunol-032712-100003. 23298207

[43] Bazer FW, Spencer TE, Johnson GA, Burghardt RC, Wu G. 2009. Comparative aspects of implantation. Reproduction 138:195–209. doi:10.1530/REP-09-0158. 19502456

[44] Asai K. 2017. Anti-sigma factor-mediated cell surface stress responses in Bacillus subtilis. Genes Genet Syst 92:223–234. doi:10.1266/ggs.17-00046. 29343670

[45] Ata B, Yildiz S, Turkgeldi E, Brocal VP, Dinleyici EC, Moya A, Urman B. 2019. The endobiota study: comparison of vaginal, cervical and gut microbiota between women with stage 3/4 endometriosis and healthy controls. Sci Rep 9:2204. doi:10.1038/s41598-019-39700-6. 30778155 PMC6379373

[46] Newman ME. 2006. Modularity and community structure in networks. Proc Natl Acad Sci USA 103:8577–8582. doi:10.1073/pnas.0601602103. 16723398 PMC1482622

[47] Boulangé CL, Neves AL, Chilloux J, Nicholson JK, Dumas M-E. 2016. Impact of the gut microbiota on inflammation, obesity, and metabolic disease. Genome Med 8:42. doi:10.1186/s13073-016-0303-2. 27098727 PMC4839080

[48] Choi Y, Kwon Y, Kim D-K, Jeon J, Jang SC, Wang T, Ban M, Kim M-H, Jeon SG, Kim M-S, Choi CS, Jee Y-K, Gho YS, Ryu SH, Kim Y-K. 2015. Gut microbe-derived extracellular vesicles induce insulin resistance, thereby impairing glucose metabolism in skeletal muscle. Sci Rep 5:15878. doi:10.1038/srep15878. 26510393 PMC4625370

[49] Raimundo J, Reis CMG, Ribeiro MM. 2018. Rapid, simple and potentially universal method for DNA extraction from Opuntia spp. fresh cladode tissues suitable for PCR amplification. Mol Biol Rep 45:1405–1412. doi:10.1007/s11033-018-4303-8. 30109548

[50] Magoč T, Salzberg SL. 2011. FLASH: fast length adjustment of short reads to improve genome assemblies. Bioinformatics 27:2957–2963. doi:10.1093/bioinformatics/btr507. 21903629 PMC3198573

[51] Rognes T, Flouri T, Nichols B, Quince C, Mahé F. 2016. VSEARCH: a versatile open source tool for metagenomics. PeerJ 4:e2584. doi:10.7717/peerj.2584. 27781170 PMC5075697

[52] Edgar RC. 2013. UPARSE: highly accurate OTU sequences from microbial amplicon reads. Nat Methods 10:996–998. doi:10.1038/nmeth.2604. 23955772

[53] Dixon P. 2003. VEGAN, a package of R functions for community ecology. J Veg Sci 14:927–930. doi:10.1111/j.1654-1103.2003.tb02228.x.

[54] Brunson JC. 2020. ggalluvial: layered grammar for alluvial plots. Joss 5:2017. doi:10.21105/joss.02017. 36919162 PMC10010671

[55] Friedman J, Alm EJ. 2012. Inferring correlation networks from genomic survey data. PLoS Comput Biol 8:e1002687. doi:10.1371/journal.pcbi.1002687. 23028285 PMC3447976

[56] Shannon P, Markiel A, Ozier O, Baliga NS, Wang JT, Ramage D, Amin N, Schwikowski B, Ideker T. 2003. Cytoscape: a software environment for integrated models of biomolecular interaction networks. Genome Res 13:2498–2504. doi:10.1101/gr.1239303. 14597658 PMC403769

[57] Bastian M, Heymann S, Jacomy M. 2009. Gephi: an Open Source Software for Exploring and Manipulating Networks. Proceedings of the International AAAI Conference on Web and Social Media 3(1):361–362. doi:10.1609/icwsm.v3i1.13937.

[58] Wemheuer F, Taylor JA, Daniel R, Johnston E, Meinicke P, Thomas T, Wemheuer B. 2020. Tax4Fun2: prediction of habitat-specific functional profiles and functional redundancy based on 16S rRNA gene sequences. Environmental Microbiome 15:11. doi:10.1186/s40793-020-00358-7. 33902725 PMC8067651

[59] Parks DH, Tyson GW, Hugenholtz P, Beiko RG. 2014. STAMP: statistical analysis of taxonomic and functional profiles. Bioinformatics 30:3123–3124. doi:10.1093/bioinformatics/btu494. 25061070 PMC4609014

[60] Yi L, Pimentel H, Bray NL, Pachter L. 2018. Gene-level differential analysis at transcript-level resolution. Genome Biol 19:53. doi:10.1186/s13059-018-1419-z. 29650040 PMC5896116

[61] Karlsson FH, Tremaroli V, Nookaew I, Bergström G, Behre CJ, Fagerberg B, Nielsen J, Bäckhed F. 2013. Gut metagenome in European women with normal, impaired and diabetic glucose control. Nature 498:99–103. doi:10.1038/nature12198. 23719380

[62] Karlsson FH, Fåk F, Nookaew I, Tremaroli V, Fagerberg B, Petranovic D, Bäckhed F, Nielsen J. 2012. Symptomatic atherosclerosis is associated with an altered gut metagenome. Nat Commun 3:1245. doi:10.1038/ncomms2266. 23212374 PMC3538954

[63] Luo R, Liu B, Xie Y, Li Z, Huang W, Yuan J, He G, Chen Y, Pan Q, Liu Y, Tang J, Wu G, Zhang H, Shi Y, Liu Y, Yu C, Wang B, Lu Y, Han C, Cheung DW, Yiu S-M, Peng S, Xiaoqian Z, Liu G, Liao X, Li Y, Yang H, Wang J, Lam T-W, Wang J. 2012. SOAPdenovo2: an empirically improved memory-efficient short-read de novo assembler. Gigascience 1:18. doi:10.1186/2047-217X-1-18. 23587118 PMC3626529

[64] Qin N, Yang F, Li A, Prifti E, Chen Y, Shao L, Guo J, Le Chatelier E, Yao J, Wu L, Zhou J, Ni S, Liu L, Pons N, Batto JM, Kennedy SP, Leonard P, Yuan C, Ding W, Chen Y, Hu X, Zheng B, Qian G, Xu W, Ehrlich SD, Zheng S, Li L. 2014. Alterations of the human gut microbiome in liver cirrhosis. Nature 513:59–64. doi:10.1038/nature13568. 25079328

[65] Li J, Jia H, Cai X, Zhong H, Feng Q, Sunagawa S, Arumugam M, Kultima JR, Prifti E, Nielsen T, Juncker AS, Manichanh C, Chen B, Zhang W, Levenez F, Wang J, Xu X, Xiao L, Liang S, Zhang D, Zhang Z, Chen W, Zhao H, Al-Aama JY, Edris S, Yang H, Wang J, Hansen T, Nielsen HB, Brunak S, Kristiansen K, Guarner F, Pedersen O, Doré J, Ehrlich SD, Bork P, Wang J, MetaHIT Consortium. 2014. An integrated catalog of reference genes in the human gut microbiome. Nat Biotechnol 32:834–841. doi:10.1038/nbt.2942. 24997786

[66] Zhu W, Lomsadze A, Borodovsky M. 2010. Ab initio gene identification in metagenomic sequences. Nucleic Acids Res 38:e132–e132. doi:10.1093/nar/gkq275. 20403810 PMC2896542

[67] Fu L, Niu B, Zhu Z, Wu S, Li W. 2012. CD-HIT: accelerated for clustering the next-generation sequencing data. Bioinformatics 28:3150–3152. doi:10.1093/bioinformatics/bts565. 23060610 PMC3516142

[68] Feng Q, Liang S, Jia H, Stadlmayr A, Tang L, Lan Z, Zhang D, Xia H, Xu X, Jie Z, Su L, Li X, Li X, Li J, Xiao L, Huber-Schönauer U, Niederseer D, Xu X, Al-Aama JY, Yang H, Wang J, Kristiansen K, Arumugam M, Tilg H, Datz C, Wang J. 2015. Gut microbiome development along the colorectal adenoma–carcinoma sequence. Nat Commun 6:6528. doi:10.1038/ncomms7528. 25758642

[69] Mortazavi A, Williams BA, McCue K, Schaeffer L, Wold B. 2008. Mapping and quantifying mammalian transcriptomes by RNA-Seq. Nat Methods 5:621–628. doi:10.1038/nmeth.1226. 18516045 PMC13303166

[70] Pertea M, Pertea GM, Antonescu CM, Chang T-C, Mendell JT, Salzberg SL. 2015. StringTie enables improved reconstruction of a transcriptome from RNA-seq reads. Nat Biotechnol 33:290–295. doi:10.1038/nbt.3122. 25690850 PMC4643835

[71] Liao Y, Smyth GK, Shi W. 2014. featureCounts: an efficient general purpose program for assigning sequence reads to genomic features. Bioinformatics 30:923–930. doi:10.1093/bioinformatics/btt656. 24227677

[72] Love MI, Huber W, Anders S. 2014. Moderated estimation of fold change and dispersion for RNA-seq data with DESeq2. Genome Biol 15:550. doi:10.1186/s13059-014-0550-8. 25516281 PMC4302049

[73] Wu T, Hu E, Xu S, Chen M, Guo P, Dai Z, Feng T, Zhou L, Tang W, Zhan L, Fu X, Liu S, Bo X, Yu G. 2021. clusterProfiler 4.0: A universal enrichment tool for interpreting omics data. Innovation (Camb) 2:100141. 34557778 10.1016/j.xinn.2021.100141 PMC8454663

[74] Ietswaart R, Gyori BM, Bachman JA, Sorger PK, Churchman LS. 2021. GeneWalk identifies relevant gene functions for a biological context using network representation learning. Genome Biol 22:55. doi:10.1186/s13059-021-02264-8. 33526072 PMC7852222

[75] De Meo P, Ferrara E, Fiumara G, Provetti A. 2011. Generalized Louvain method for community detection in large networks, p 88–93. *In* Proceedings of the 11th International Conference On Intelligent Systems Design And Applications. IEEE, Spain. doi:10.1109/ISDA.2011.6121636.

