# Supplementary figures and images for: Interaction between Microbes and Host in Sow Vaginas in Early Pregnancy
Source: mSystems. 2023 Feb 7;8(2):e01192-22. doi: 10.1128/msystems.01192-22 (PMC10134864; doi:10.1128/msystems.01192-22)

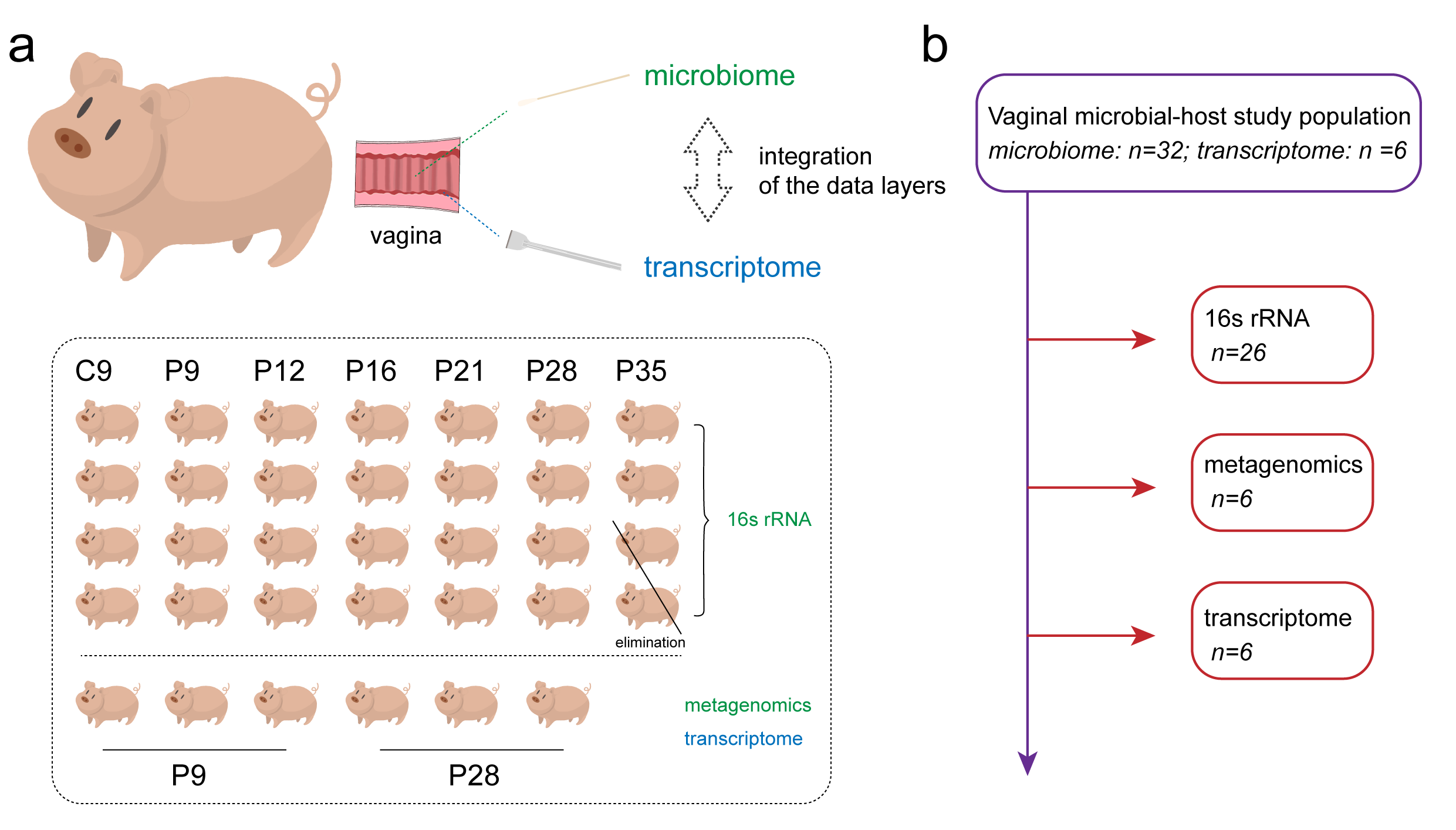

Supplement: FIG S1 [file msystems.01192-22-s0001.tif]

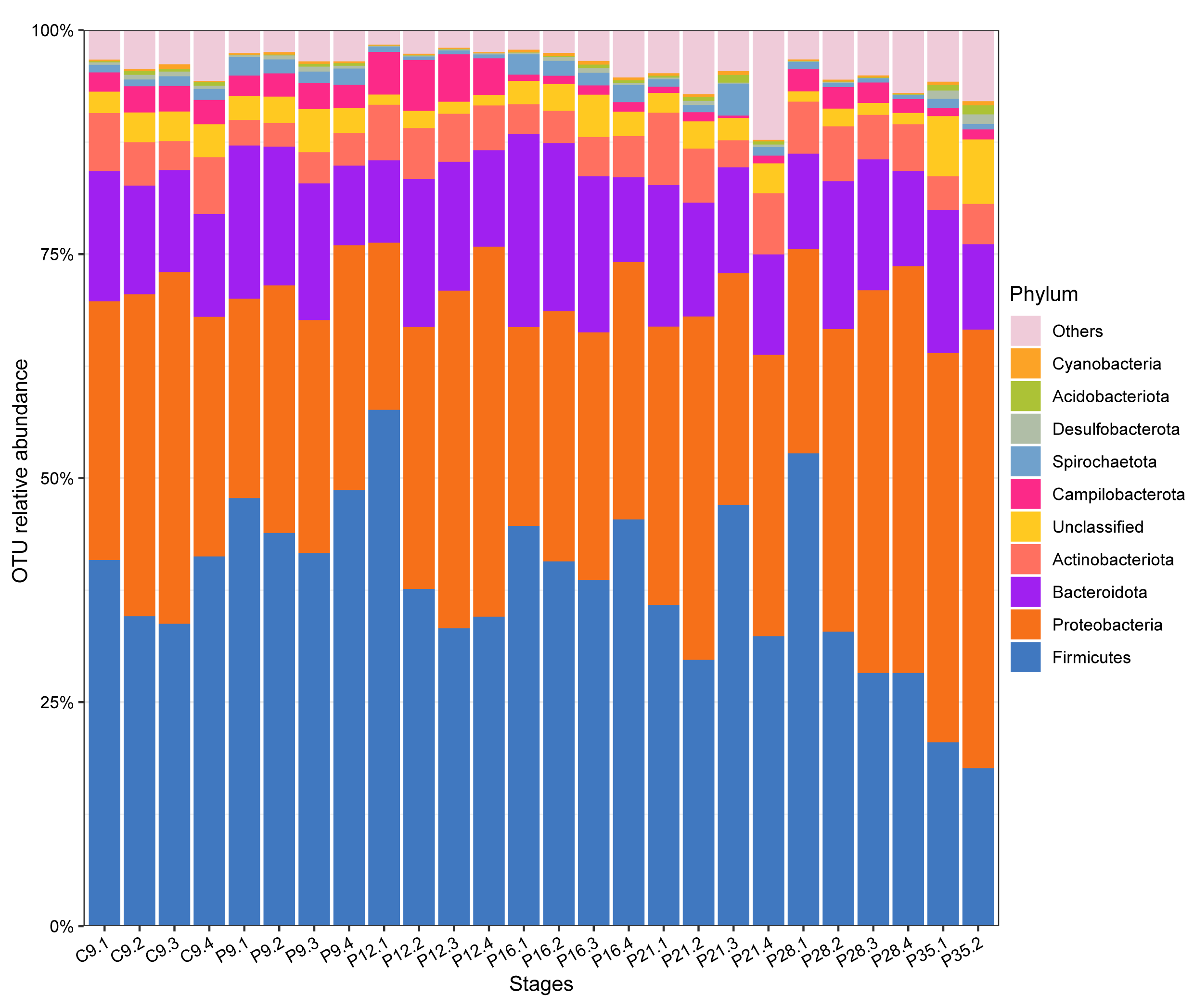

Supplement: FIG S2 [file msystems.01192-22-s0002.tif]

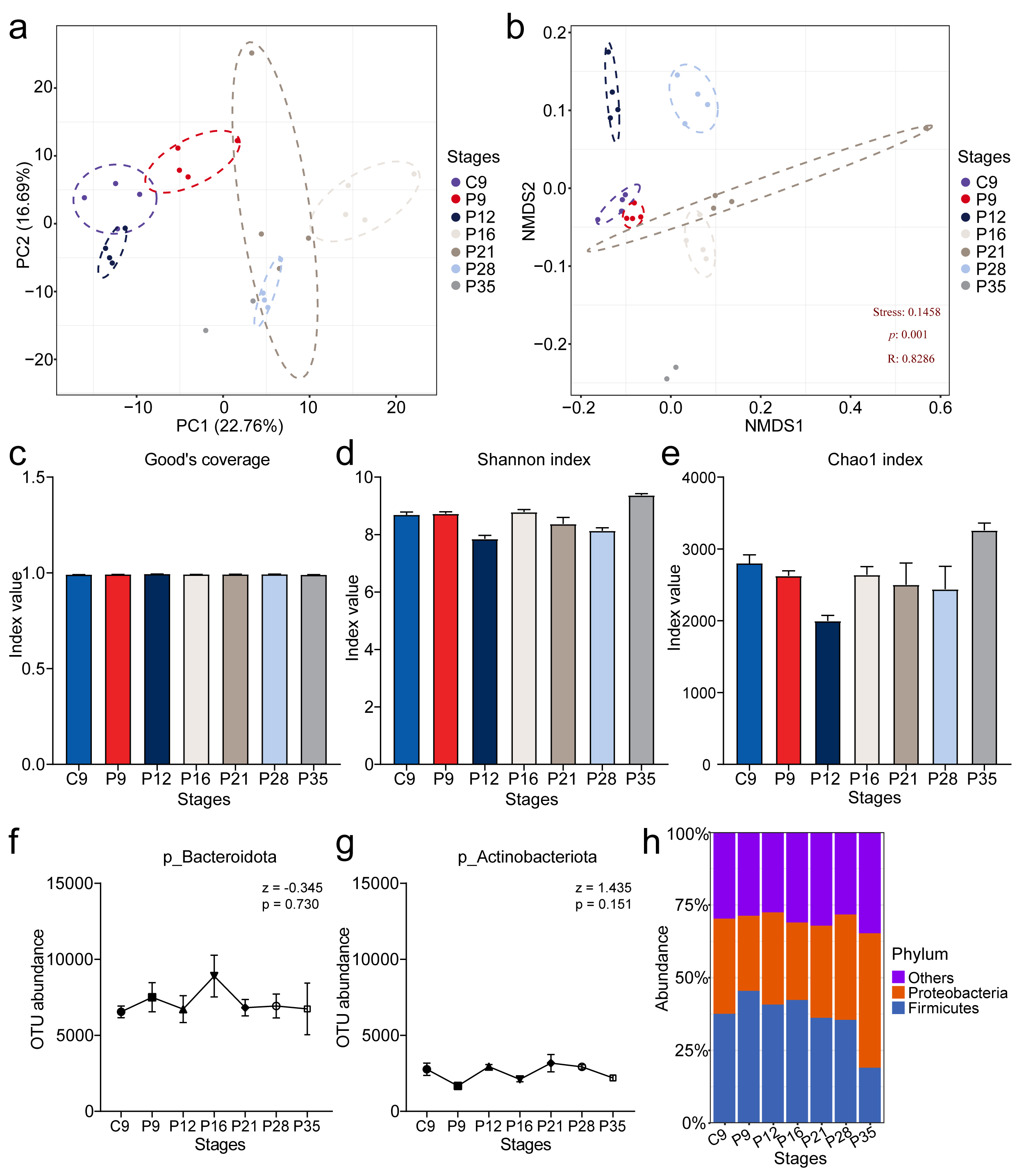

Supplement: FIG S3 [file msystems.01192-22-s0003.tif]

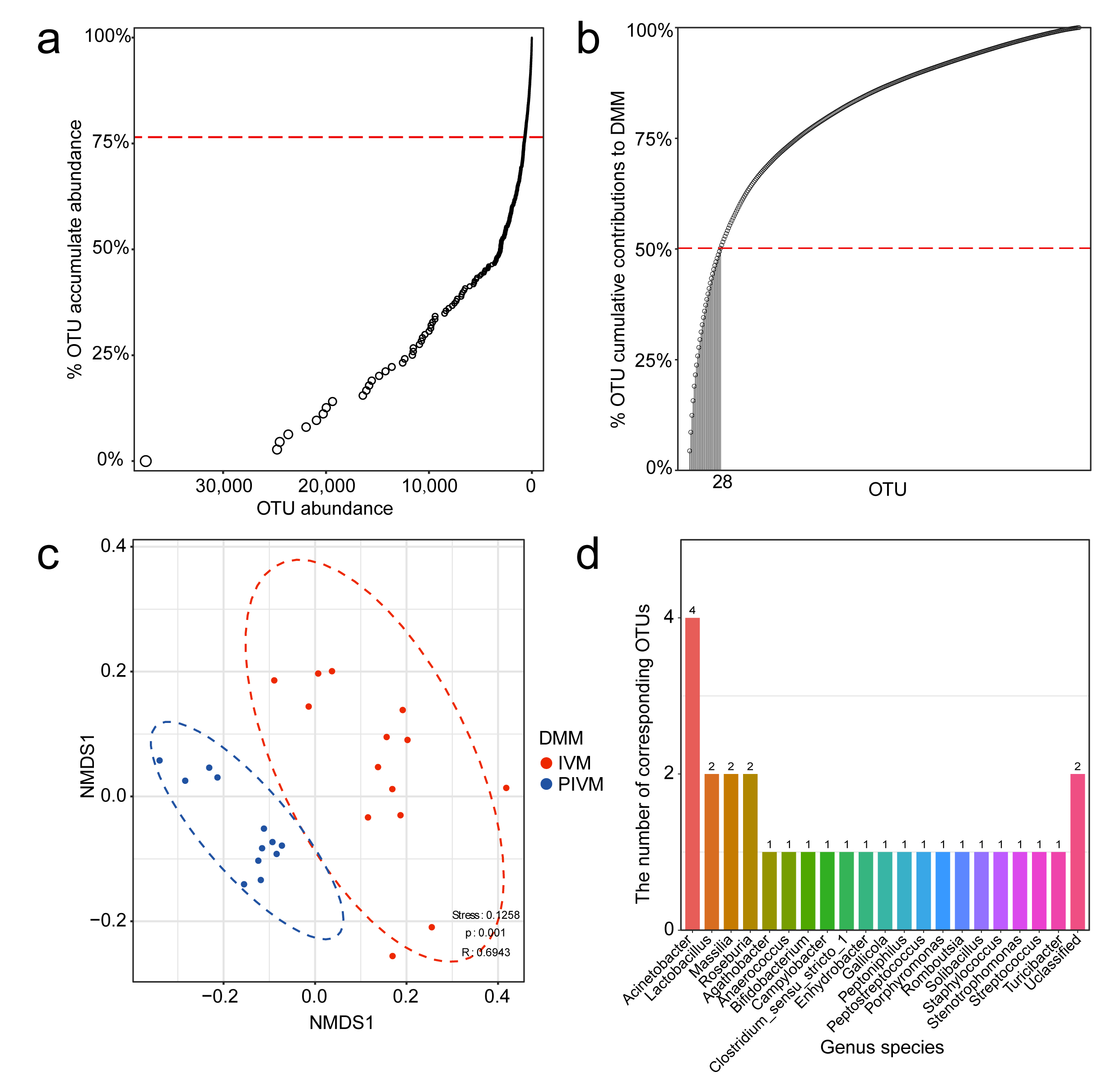

Supplement: FIG S4 [file msystems.01192-22-s0004.tif]

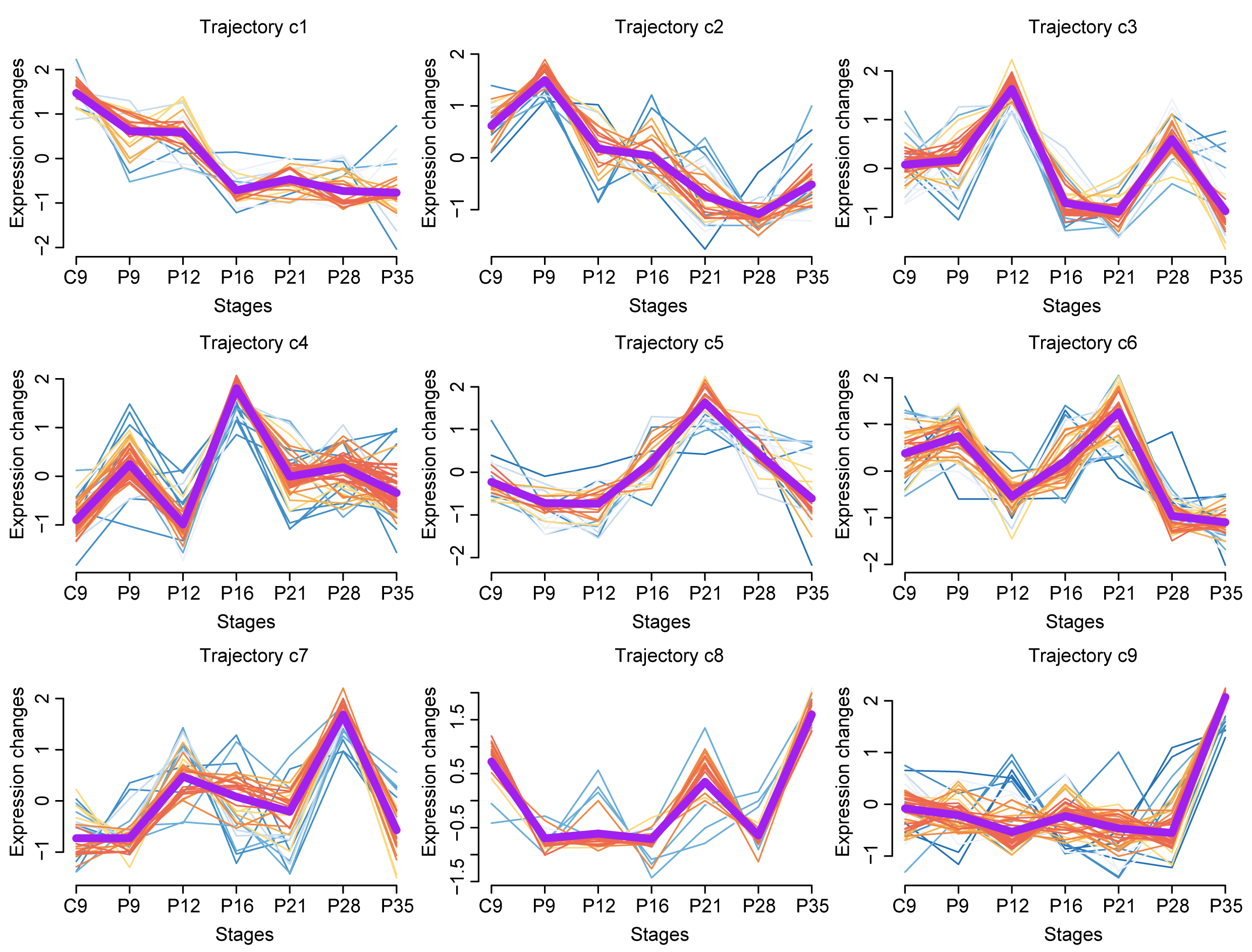

Supplement: FIG S5 [file msystems.01192-22-s0005.tif]

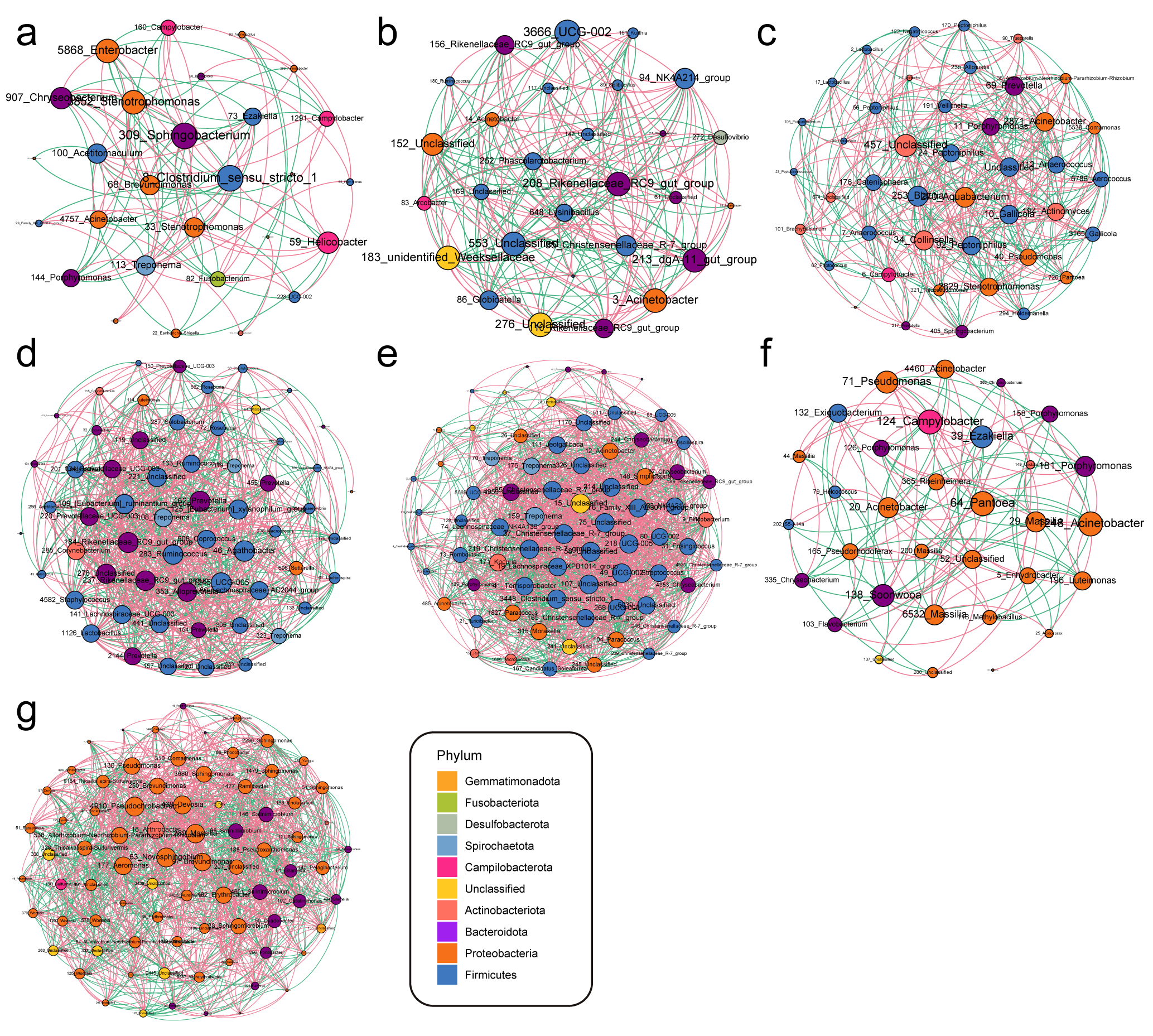

Supplement: FIG S6 [file msystems.01192-22-s0006.tif]

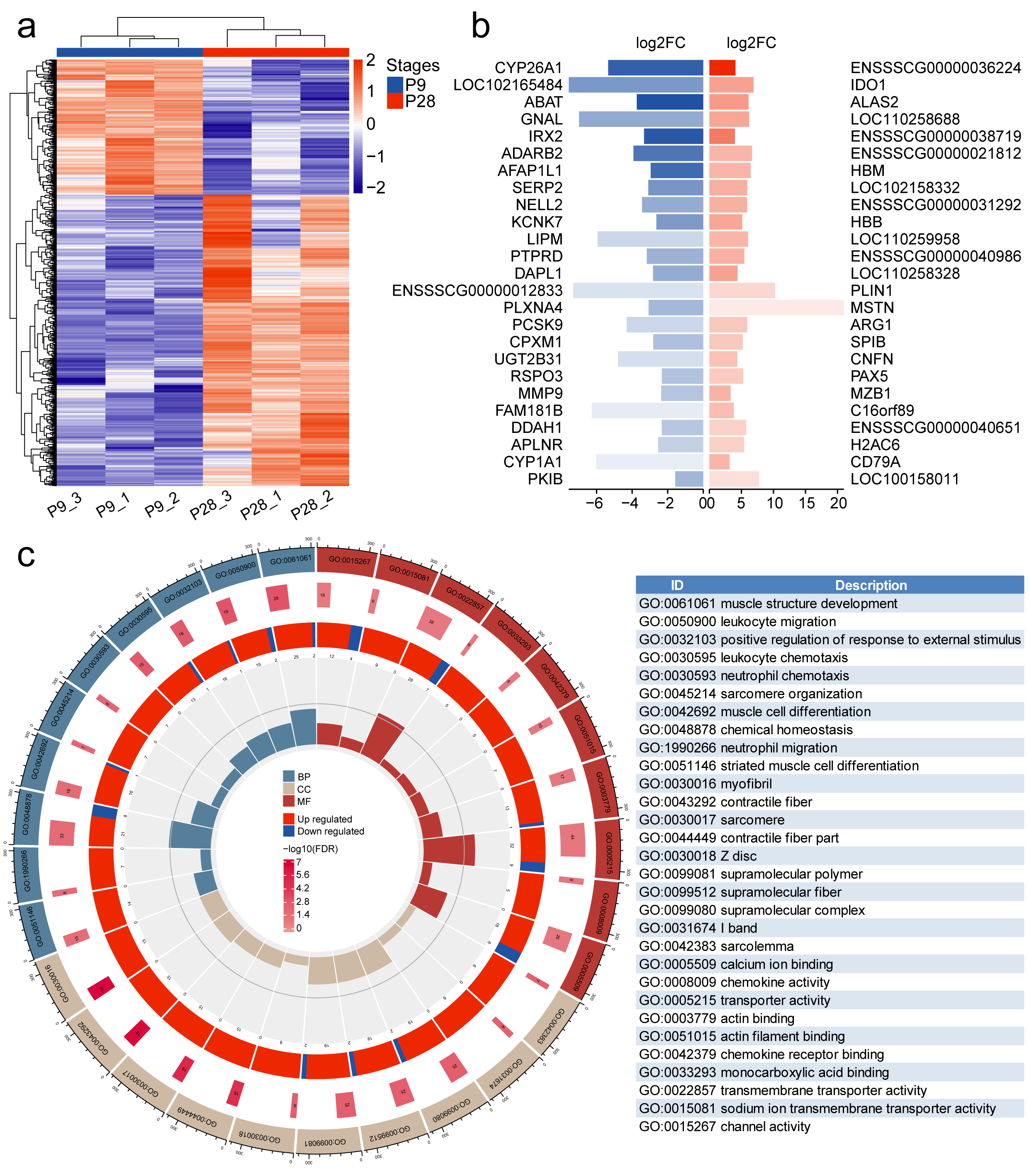

Supplement: FIG S7 [file msystems.01192-22-s0007.tif]

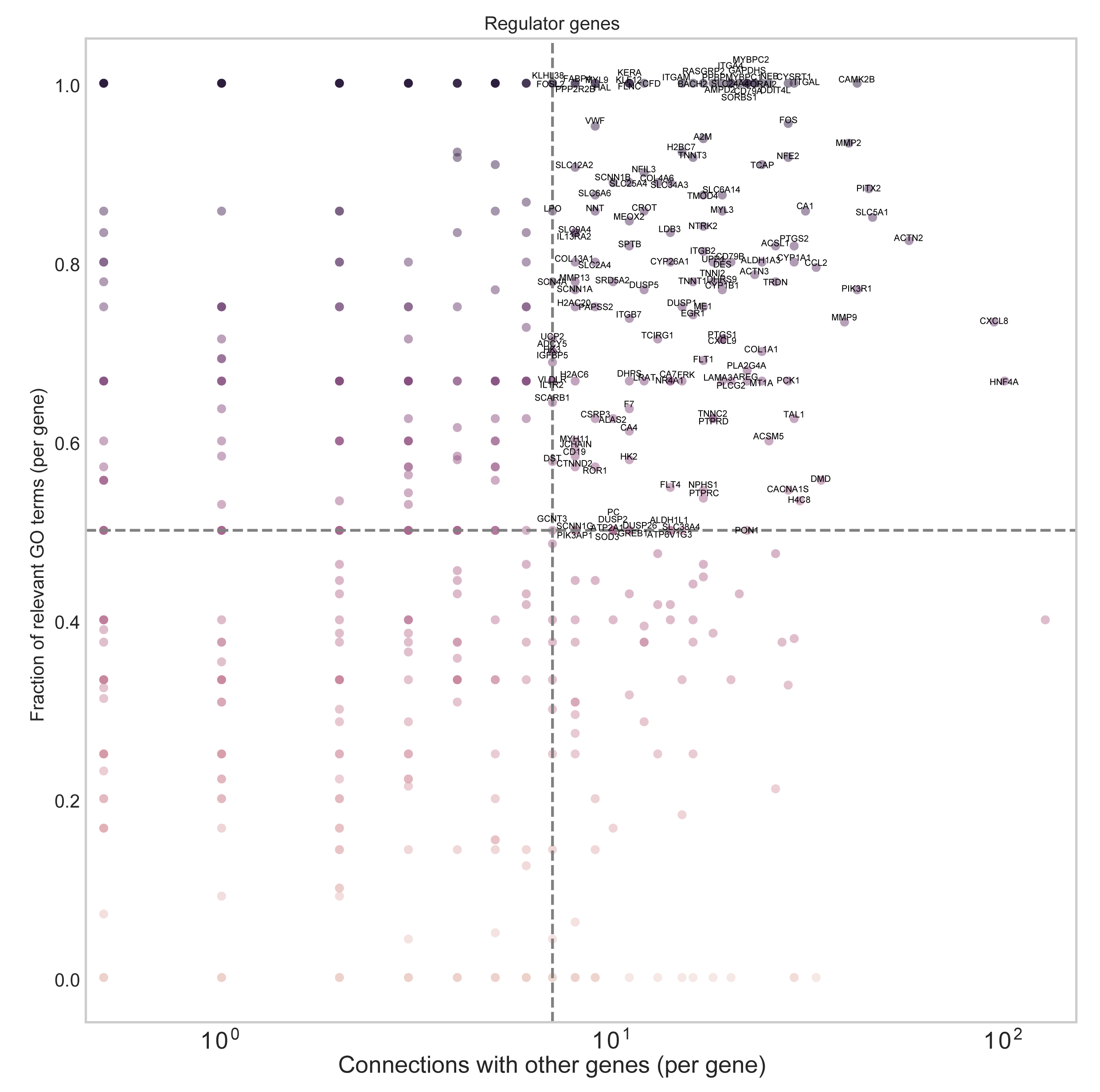

Supplement: FIG S8 [file msystems.01192-22-s0008.tif]

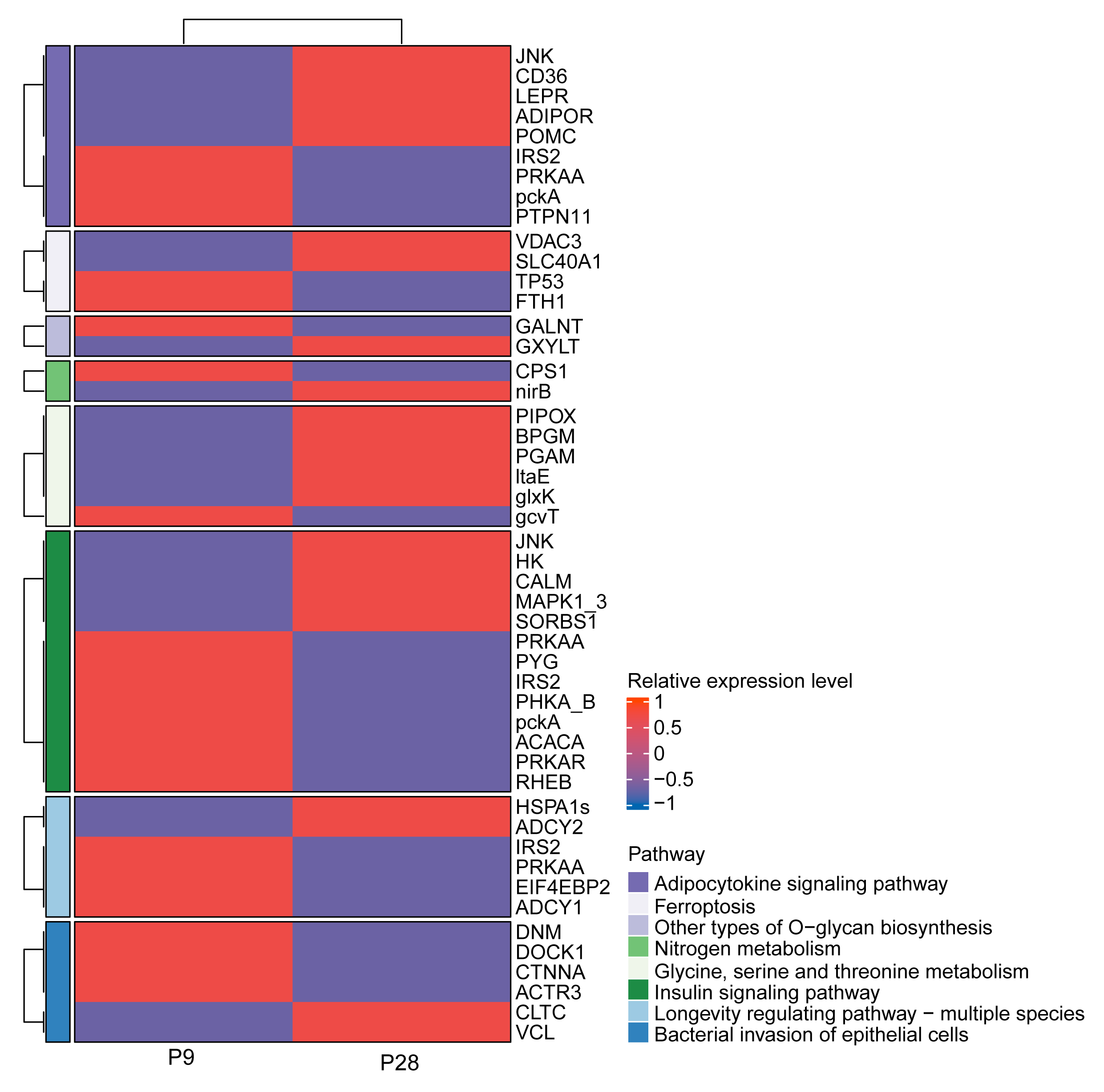

Supplement: FIG S9 [file msystems.01192-22-s0009.tif]

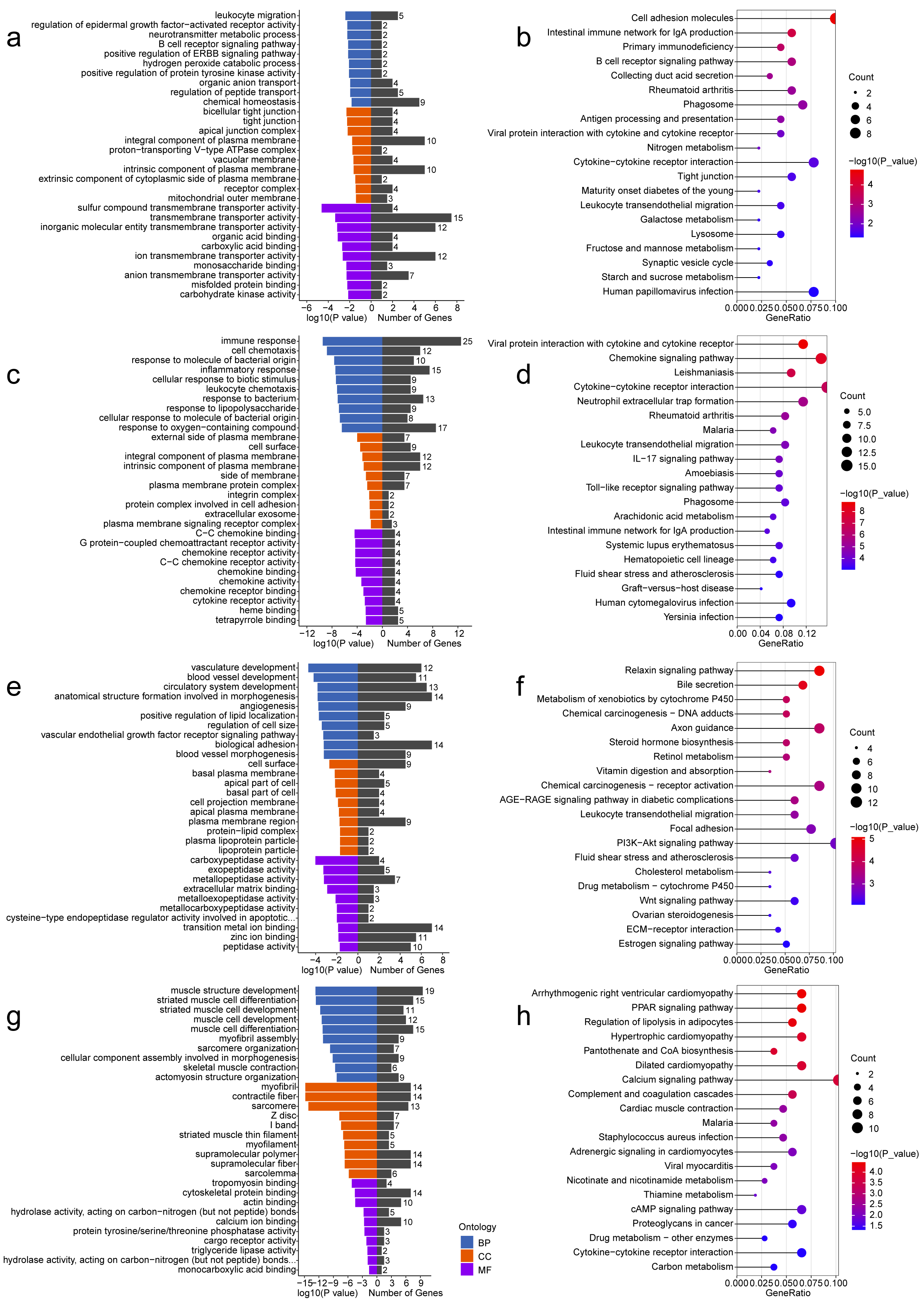

Supplement: FIG S10 [file msystems.01192-22-s0010.tif]
